# Leveraging public data to offer online inquiry opportunities

**DOI:** 10.1002/ece3.6706

**Published:** 2020-09-24

**Authors:** Seth K. Thompson, Catherine Kirkpatrick, Maxwell Kramer, Sehoya Cotner

**Affiliations:** ^1^ Biology Teaching and Learning University of Minnesota‐Twin Cities Minneapolis MN USA

**Keywords:** data‐driven inquiry, distance learning, global change ecology, remote instruction

## Abstract

Inquiry activities have become increasingly common in *Ecology and Evolution* courses, but the rapid shift to remote instruction for many faculty members in response to the COVID‐19 pandemic has created new challenges for maintaining these student‐centered activities in a distance learning format. Moving forward, many instructors will be asked to create flexible course structures that allow for a mix of different teaching modalities and will be looking for resources to support student inquiry in both online and in‐person settings. Here, we propose the use of data‐driven inquiry activities as a flexible option for offering students experiences to build career‐relevant skills and learn fundamental ecological concepts. We share lessons learned from our experiences teaching a two‐semester course‐based research experience in global change ecology that leverages publicly available datasets to engage students in broadly relevant scientific inquiry.

## Graphical abstract

Inquiry activities have become increasingly common in *Ecology and Evolution* courses, but the rapid shift to remote instruction for many faculty members in response to the COVID‐19 pandemic has created new challenges for maintaining these student‐centered activities in a distance learning format. Here, we propose the use of data‐driven inquiry activities as a flexible option for offering students experiences to build career‐relevant skills and learn fundamental ecological concepts. We share lessons learned from our experiences teaching a two‐semester course‐based research experience in global change ecology that leverages publicly available datasets to engage students in broadly relevant scientific inquiry.

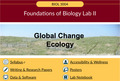

## 1 INTRODUCTION

The trajectory of instructional practices in higher education over the last decade has resulted in a shift toward more student‐centered pedagogies in *Ecology and Evolution*. Following national calls (Brewer & Smith, 2011; Olson & Riordan, 2012) for instructional reform in undergraduate education, many instructors have integrated more inquiry‐based learning opportunities and have adopted active learning into their courses. While adoption of these practices has been far from universal (Stains et al., 2018), there is strong evidence that integrating these student‐focused approaches results in better learning outcomes (Freeman et al., 2007, 2014; Theobald et al., 2020). Additionally, a growing number of instructors are integrating course‐based research experiences (CREs) into their curriculum (Auchincloss et al., 2014; Ballen, Thompson, Blum, Newstrom, & Cotner, 2018; Kirkpatrick, Schuchardt, Baltz, & Cotner, 2019; Thompson, Neill, Wiederhoeft, & Cotner, 2016) in an attempt to scale the documented benefits (Hunter, Laursen, & Seymour, 2007; Lopatto, 2007; Russell, Hancock, & McCullough, 2007) of research experience to a larger number of students.

The rapid shift to remote instruction has raised several challenges to maintaining these evidenced‐based instructional practices because many of these techniques were designed with an in‐person classroom in mind. For example, facilitating student interactions in a way that promotes active learning can be challenging in an online setting. Cultivating a strong course‐based community and replacing research and/or laboratory experiences for students have also proven to be major challenges for instructors during the rapid shift to remote instruction. Given the continued impacts of the COVID‐19 public health crisis, many instructors will be asked to develop online instructional materials for their courses in future terms; in particular, this fall may necessitate a high amount of instructional flexibility. With the nature of many faculty appointment structures (such as 9‐month contracts and limited time to develop course materials), many of our colleagues who are charged with facilitating inquiry online will be seeking resources that are easy to implement.

Computational ecology and the use of simulation models have become important tools for studying ecosystem‐scale questions within ecology (Hogan & Weathers, 2003; Weathers et al., 2016; Winslow et al., 2016). For example, the use of simulation models has become a critical tool for exploring the potential responses of ecosystems to a rapidly changing climate (Farrell et al., 2020; Hansen, Read, Hansen, & Winslow, 2017). The concurrent rise of large publicly available datasets has offered instructors the opportunity to integrate more data‐driven inquiry into their ecology classes by using these datasets to promote student development of computational literacy (Farrell & Carey, 2018). We propose that data‐driven inquiry, where students use previously generated datasets to explore scientific questions they are interested in, represents an opportunity to maintain student‐driven inquiry and course‐based research experiences during remote instruction while maintaining a focus on career‐relevant skill development for students. For example, these data‐driven experiences have been associated with improved quantitative literacy (Klug, Carey, Richardson, & Darner Gougis, 2017), as well as gains in confidence working with data and data analysis skills (Carey & Gougis, 2017; Carey, Gougis, Klug, O'Reilly, & Richardson, 2015; Farrell & Carey, 2018).Therefore, facilitating opportunities for data‐driven inquiry in ecology courses has great potential for enhancing the skills needed for students to become successful ecologists in a rapidly changing field that is increasingly using large datasets to study global phenomena.

Luckily for instructors, many resources already exist for bringing data‐driven inquiry into the classroom (Farrell & Carey, 2018). For example, Project EDDIE, Macrosystems EDDIE, and the National Ecological Observatory Network (NEON) Teaching Models all host freely available materials ready for classroom use. Furthermore, by leveraging these existing resources for data‐driven inquiry combined with the growing availability of publicly available datasets in ecology (Table 1), it is possible to engage students in authentic ecological research experiences during remote instruction. In this paper, we present lessons learned from shifting a computational CRE in global change ecology to remote instruction and provide recommendations for adopters.

**Table 1:** Examples of publicly available datasets for data‐driven inquiry

Available dataset	Hosting agency	Website	Description
National Aquatic Resource Surveys	EPA	https://www.epa.gov/national‐aquatic‐resource‐surveys	National datasets capturing water quality parameters for lakes, streams, wetlands, and coastal systems
Biology and Ecosystem Datasets	USGS	https://www.usgs.gov/products/data‐and‐tools/science‐datasets	Over 10,000 different datasets from USGS Science programs. Filterable by location, year, and topic
Climate Data Online	NOAA	https://www.ncdc.noaa.gov/cdo‐web/datasets	Various climate and marine datasets, searchable through an online interface
LTER Datasets	Various‐ for example: Cedar Creek Ecosystem Science Reserve	https://www.cedarcreek.umn.edu/research/data	Data from the Cedar Creek LTER program. Includes projects related to grassland productivity, ecosystem fertilization, and many others. Other LTER sites around the nation also host publicly available datasets
State Agency Datasets	Departments of Natural Resources, Pollution Control Agencies, etc.	Vary by state‐ for example in MN https://www.pca.state.mn.us/environmental‐data	Datasets vary by state and region, but can provide local connections for students to examine locally relevant issues
DataOne repository of various data networks	DataOne network	https://www.dataone.org	Open platform for searching several different data repositories. Also includes learning materials for using large datasets
Community Science Data Programs	Various, for example: Zooniverse	https://www.zooniverse.org	Interactive platform for community‐led science projects (citizen science). Covers a variety of disciplines, including “Nature” which currently has 59 active projects
National/International Environmental Datasets	Various, for example: Australian Government Environmental Open Data; African Environment & Climate Change Data Portal	http://www.environment.gov.au/about‐us/environmental‐information‐data/open‐data https://africaclimate.opendataforafrica.org	There are a variety of data portals hosted by governmental agencies and nonprofits that explore environmental issues at national and international scales. Some include dashboards for exploring data and others have raw data readily available
Bird Migration Patterns in the Western Hemisphere	Cornell Lab of Ornithology	Ebird.org	Community generated data on bird sightings in the western hemisphere

## 2 FOUNDATIONS OF BIOLOGY AT THE UNIVERSITY OF MINNESOTA

The Foundations of Biology laboratory series is a two‐semester experience for students in the College of Biological Sciences at the University of Minnesota. This introductory series is students' first biology course experience and requires one semester of College level chemistry (or equivalent credits) prior to enrollment. Therefore, it is most typically started in the second semester or the third semester of a student's program. The laboratory series is structured as a course‐based research experience (CRE) and serves as the introduction to scientific research for most students. The first semester is broken up into two halves. In the first half, students engage in broad‐based skill‐building activities. These activities range from building basic laboratory skills like pipetting, using a spectrophotometer, and running a gel, to an introduction to computational skills such as basic data analysis and programming in R. To prepare students for future data‐driven inquiry, students complete guided skill‐building activities covering reproducible data analysis by writing basic R scripts, data visualization strategies using large, pooled student‐generated datasets, and drawing conclusions from large, public datasets. After this initial skill‐building phase, students select a specific research area to pursue. For the second half of the first semester, students engage in project‐specific training relevant to their research area. The first semester culminates in the writing of a research proposal, which students then complete in their second semester of the laboratory. A more detailed description of the course structure can be found in Kirkpatrick et al. (2019).

One of the research areas available to students is global change ecology. Started in 2018, the global change ecology research area uses data‐driven inquiry activities and large public datasets to facilitate the CRE. With an emphasis on building students' computational literacy and data skills, global change ecology encourages students to wrestle with some of the pressing ecological issues we face today. In the project‐specific training period of the first‐semester laboratory, students complete a mix of data‐exploration activities either created by instructors of the course or adapted from Project EDDIE or Macrosystems EDDIE (Appendix S1). Students are introduced to analytical tools and software that range from point and click spreadsheets to command‐line tools that use coding languages, including Excel, R, and JMP. Students build basic skills using that software and models/simulations while they explore topics such as the impacts of fertilization on global grassland ecosystems, impacts of climate change on aquatic ecosystems, and the relationship between greenhouse gases concentrations and temperature reconstructions from ice cores. Through these experiences, students learn basic data management; they are also introduced to basic statistical analysis procedures such as simple linear regression analysis, as well as more complex simulation models as a tool for studying ecosystem‐scale processes. Lastly, students use reproducible research practices, such as documenting analysis steps in written scripts, including raw data in final reports, and assessing the role of software tools in the reproducibility of their work.

In the second‐semester course, students use existing public datasets (or generate their own through combining multiple datasets) to examine research questions they have generated with the support of a postdoctoral research mentor. For example, previous students have asked questions about the impact of warming lake temperatures on walleye spawning rates or how increased nutrient loading in lakes impacts invasive species dynamics. The semester concludes with a college‐wide poster session, in which students share the results of their work with other students and faculty from throughout the college; they also write an individual research paper based on their semester‐long project.

In spring of 2020, the University of Minnesota switched to remote instruction in response to the COVID‐19 public health crisis. This switch occurred in mid‐March, corresponding to the project‐specific training portion of the first‐semester course and mid‐project completion in the second‐semester course. Below, we describe our strategy for maintaining our CRE during remote instruction and share insights from our experience to help instructors attempting to facilitate CREs in a remote setting.

## 3 STRATEGY FOR SHIFTING TO REMOTE INSTRUCTION

The overall approach for shifting to remote instruction revolved around establishing a consistent routine for students. Through a brief survey, we were able to identify that several students in the course did not have unlimited access to reliable wifi, so we decided to minimize the amount of synchronous instruction that was required for students. For students participating in the project‐specific training portion of the first‐semester course, we shifted didactic content and moved to prerecorded videos that were made available on the course page for students to view at their convenience. Teaching assistants (TAs) remained available during the scheduled course period to provide synchronous support for students needing it in an office hours type format. These sessions were intended to allow students to have real‐time feedback on their progress during the data‐driven activities. In addition to this synchronous support, we established an online discussion space that was monitored by the course instructors as well as the TAs.

In the second‐semester course, the switch to remote instruction occurred at a point in the semester where students had largely established their routine in completing their research projects with their group members. During the semester, students meet with their TA once a week for a ~2‐hr session that operates as a laboratory meeting and complete the majority of their research work outside of this time. After the switch to remote instruction, this synchronous time was replaced with a weekly video from the TA detailing the work to be completed for the week and students continued to arrange virtual time with their group mates to complete their research projects.

## 4 SUCCESSES

Overall, the use of data‐driven inquiry allowed for a relatively straightforward transition to remote instruction in comparison with the transition experienced in more laboratory‐intensive research projects. Each of the learning activities had student handouts intended to walk students through the activity and the maintenance of a synchronous office hours period allowed students to check in with a TA in a similar way to the in‐person instructional model. This allowed us to maintain the core structure of the project‐specific training without having to eliminate any of the usual learning activities. The key to the success of transitioning the data‐driven materials was the highly detailed written guides for students. Modeling the design of our own activities after the level of detail provided in the Project EDDIE student handouts, we were able to create materials that students could complete in a self‐guided fashion. In combination with the online discussion boards, this allowed students to work on the material in a highly asynchronous manner while still feeling well supported.

Another tool that proved to be very useful during the switch to remote instruction was the use of RStudio Cloud for all of the activities that required coding in R. This online platform for delivery of R‐based content allows instructors to preload datafiles, scripts, software packages, and other materials to help reduce the initial learning curve associated with programming for many students. By using RStudio Cloud, students no longer needed to install any software on their local computers or navigate idiosyncratic local file structures, two large sources of frustration that hinder novice R users' learning experiences. It also allows instructors to remotely view and even edit students' code within RStudio Cloud to help troubleshoot code errors. This tool allowed us to retain an “introduction to ecosystem simulation models” activity and we would highly recommend it for others pursuing R‐based activities in a remote instruction environment.

Finally, we had good success transitioning our end‐of‐semester poster session into a distanced format. We offered students two options for presenting their research posters, an asynchronous option using VoiceThread and a synchronous option using Zoom with breakout rooms. A relatively small fraction of students chose to participate in the synchronous modality, but from an instructional perspective it more closely matched the in‐person experience for students, and those who participated reported a better experience with it than the asynchronous VoiceThread poster sessions.

## 5 REMAINING CHALLENGES

One of the biggest challenges during the abrupt switch to remote instruction was maintaining a sense of classroom community. Particularly in the second‐semester course where all synchronous instruction was limited, students reported a sense of disconnect from the course and a desire for a set meeting time to maintain group cohesion and progress. This suggests that instructors may want to aim for at least a small amount of synchronous instruction to help students maintain a connection with the course and their classmates, particularly in courses that incorporate group work. Along these same lines, we observed a much stronger need for formalized formative assessment opportunities for students during remote instruction. In a traditional classroom setting, there are several opportunities for instructors to provide informal formative assessments through casual feedback, short conversations, and other check‐ins. However, in our switch to remote instruction, we did not anticipate the degree to which this formative feedback would be lost to students; thus, we encourage instructors to be purposeful in integrating opportunities to provide low‐stakes, consistent formative feedback for students. This is especially true as students encounter frustrations while working through data‐intensive and computational activities that may feel foreign or intimidating to them. Real‐time feedback, very clear instructions, and structured support are critical for mitigating feelings of isolation and to help students remain engaged in the course activities.

A second major theme that arose from our experience was that transparency in decision‐making and communication is key for cultivating classroom community and promoting student success. Particularly in a situation where the format of the course changed rapidly, students wanted to understand how their course experience was going to change and the reasoning for the decisions behind it. While this can present a challenge for instructors because we are often making decisions in near real time, clearly communicating with students about what expectations have changed and which have remained consistent is critical. Students were hesitant to reach out when they encountered uncertainty or problems, an important reminder that instructors should be as proactive as possible in communicating about assignments and activities. For example, weekly emails from the instructor that detail any changes to the course content for the week and explain the need for those changes may help students keep track of expectations and ease feelings of being overwhelmed. We also found that transparency in grading was critical for students as well. Because students lacked easy opportunities to ask instructors for clarification on assignments, there was an increased importance for clearly communicating to students why points were taken off and how they could improve their work. We found that the larger assignments that we had developed clear rubrics for were better for clearly indicating grading decisions and would encourage instructors to integrate grading rubrics into as many of their assignments as possible. Lastly, we found that the tone used in providing students grading feedback was important as well. Without the ability to clarify comments in real time during in‐person instruction, some students reported that feedback received during remote instruction was overly harsh, potentially driven by the fact that they were not as likely to get verbal feedback from their TA on these same assignments. This further emphasized the need for clear, supportive, and transparent communication with students throughout the remote instructional period.

Lastly, we found that during remote instruction it was critical to maintain a clear and consistent course structure that allowed students to establish their own routines. Without the regular structure of the in‐person classes, many students reported struggling with staying on top of course work and staying motivated. Even relatively small shifts in due dates or small alterations to assignments seemed to create more struggle for students than in a typical semester. The more we could create a consistent routine for students (i.e., posting all materials by Monday morning, having all assignments due on the same day, keeping consistent virtual office hours) the more the students seemed to understand what was happening in the course and the easier it was for them to complete their work.

## 6 REFLECTIONS AND IMPLICATIONS

In this paper, we share reflections on our experience shifting a computational CRE in global change ecology to a remote instruction format. We found that data‐driven inquiry activities provided for a relatively easy switch in instructional modality, which allowed for a resilience in the course structure that was much higher than other laboratory courses.

Based on this experience, we believe that computational ecology represents a viable option for running a CRE during remote instruction. Additionally, computational literacy and data processing align with the skills needed for ecology students in the 21st century and represent important learning in all instructional formats. For instructors planning for ecology laboratory courses, we suggest integrating data‐driven inquiry into your course structures to provide a flexible option for students in future semesters. For instructors wanting to integrate data activities into ecology courses, there are developed lessons that can be integrated into any introductory ecology course (Farrell & Carey, 2018) and we welcome inquiries about our own experience with data‐driven inquiry activities. We have provided a number of resources from our own course here as appendices, including sample course schedules, sample assignments, and rubrics.

## CONFLICT OF INTEREST

All authors declare that they do not have any conflict of interest.

## AUTHOR CONTRIBUTIONS

**Seth K. Thompson:** Conceptualization (lead); Project administration (equal); Writing‐original draft (lead). **Catherine Kirkpatrick:** Conceptualization (supporting); Funding acquisition (equal); Project administration (equal); Writing‐review & editing (equal). **Maxwell Kramer:** Conceptualization (supporting); Project administration (equal); Writing‐review & editing (equal). **Sehoya Cotner:** Conceptualization (supporting); Funding acquisition (equal); Writing‐review & editing (equal).

## Supporting information

Appendix S1‐S8

## Data Availability

The contents of this paper are reflections of the experience of the authors during the instructional period described and do not use underlying data sources.
