# Supplementary material for: Leveraging public data to offer online inquiry opportunities
Source: Ecol Evol. 2020 Sep 24;10(22):12555–60. doi: 10.1002/ece3.6706 (PMC7679546; doi:10.1002/ece3.6706)
Supplement: Supplementary file 1 — Appendix S1‐S8 [file ECE3-10-12555-s001.pdf]

Table of Contents

Appendix 1: Example schedule from project specific training in global change ecology

Appendix 2: Example of introductory data activity that uses published data from the Nutrient Network

Appendix 3: Example schedule from the second semester course

Appendix 4: Example linear regression assignment from second semester course

Appendix 5: Research paper guidelines (used in multiple research areas)

Appendix 6: Research Paper Rubric

Appendix 7: Poster guidelines (including rubrics)

Appendix 8: Example Student Projects

Appendix 1: Example schedule from project specific training in global change ecology

Week	Day	Date	Lab Activities	Lab Assignments	Project Activities/Assignments
7	M/Tu	3/2 & 3/3	Ex 8 Introduction to Global Change Ecology<br>-Research Mentor Introduction<br>-Global Research Networks	· *No pre-lab questions for Ex. 8*<br>· GMO post-lab assignment due<br>· Lab notebook check #2 (Ex. 5-7) due	· Introduction to the project<br>· Organize jigsaw reading (assign readings during lab)
	W/Th	3/4 & 3/5	Ex 9 Introduction to Working with Data<br>-Forms and Formats<br>-Introduction to Global Climate (NOAA activity)	·Ex 9: pre-lab questions due	
	M/Tu	3/9 & 3/10	**NO LABS – SPRING BREAK**		
	W/Th	3/11 & 3/12			
8	M/Tu	3/16 & 3/17	Class cancelled due to Coronavirus response		
	W/Th	3/18 & 3/19	No new work. Catch up on Ex. 8 & 9. Prepare for online instruction.	· Ex 9 post-lab assignment due (Friday)<br>· Ex 8 post-lab assignment due (Friday)	
9	M/Tu	3/23 & 3/24	Ex. 10 Ecosystem Metabolism Day 1 (Project EDDIE)	· Ex 10 pre-lab assignment due at regular lab time	− Public data set assignment assigned
	W/Th	3/25 & 3/26	Ex. 10 Ecosystem Metabolism Day 2 (Project EDDIE)<br>-Jigsaw Activity		**· Jigsaw reading assignment due**
10	M/Tu	3/30 & 3/31	Ex. 11 Lake Ice Module- Day 1 (Project EDDIE)<br>-Brainstorming Research Questions	· Ex 11 pre-lab assignment due	· Jigsaw paper discussion in "lab"<br>·brainstorm research question ideas during lab this week

	W/Th	4/1 & 4/2	Ex. 11 Lake Ice Module- Day 2 (Project EDDIE)<br>-Refining Research Questions	· Ex 10 post-lab assignment due	**- Public Data Set Assignment Due Friday 4/3**<br>**- Submit Research Question to assignment by Monday 4/6**
11	M/Tu	4/6 & 4/7	-Peer Review Research Questions<br>-Introduction to Searching the Literature		· Evaluating Research Question Idea – give peer feedback by Thurs 4/9<br>Assign Annotated Bibliography
	W/Th	4/8 & 4/9	Ex. 12 Regression Models in Ecology<br>- Multiple Regression Models and Structural Equation Models	Ex 12 pre-lab reading (no assignment due)<br>Ex 11 post-lab assignment due	**Evaluating Research Question Idea assignment due today**
12	M/Tu	4/13 & 4/14	Ex. 13 Fertilization in Grasslands	Lab Notebook Check #3 (Ex 8-11)<br>Ex 13 pre-lab assignment due	**· Submit working draft of research question on Canvas– due today**
	W/Th	4/15 & 4/16		Ex 12 post-lab assignment due	
13	M/Tu	4/20 & 4/21	Ex. 14 Climate Change and Lake Temp (Macrosystems EDDIE)	Ex 14 pre-lab assignment due	
	W/Th	4/22 & 4/23		Ex 13 post-lab assignment due	· Annotated bibliography due
14	M/Tu	4/27 & 4/28	Lightning Talks<br>Writing Workshop- ask question, get feedback from your TA	Lighting Talk Due	
	W/Th	4/29 & 4/30		Ex 14 post-lab assignment due<br>Lab notebook check #4 (Ex.12-15), notebook ready by midnight 5/4 (all lab sections)	· Final written proposal due by midnight 5/4 (all lab sections)
15	M/Tu	5/4 & 5/5	**NO LABS**		
	W/Th	5/6 & 5/7			

*Certain exercises may require more or less time than expected. We may need to change the laboratory schedule to accommodate this. If we do change the schedule, we will notify you in time for you to plan accordingly.

Appendix 2: Example of introductory data activity that uses published data from the Nutrient Network

Exercise 3
Analyzing Primary Production Across Space and Time

To do before lab:

- Read through this entire document.
- Look over and answer pre-lab questions from the student lab manual
- Install JMP if you would like to do the data analysis on your own computer. The software can be found at https://it.umn.edu/jmp
- Read the following papers, which are available on the Canvas site:
    - Fay, P. A., Prober, S. M., Harpole, W. S., Knops, J. M., Bakker, J. D., Borer, E. T., ... & Adler, P. B. (2015). Grassland productivity limited by multiple nutrients. *Nature Plants*, *1*, 15080.
    - Elser, J. J., Bracken, M. E. S., Cleland, E. E., Gruner, D. S., Harpole, W. S., Hillebrand, H., … Smith, J. E. (2007). Global analysis of nitrogen and phosphorus limitation of primary producers in freshwater, marine and terrestrial ecosystems. *Ecology Letters*, *10*(12), 1135–1142.
- Answer the pre-lab questions (Canvas assignment).

GOALS/SKILLS

After completing this experiment, you will be able to:

- **Become familiar with the functional groups of grassland plants (forbs, legumes, grasses) and their ecological niches.**
- **Understand the source of primary productivity data.**
- **Create a graph showing the relationship between primary production and nutrient availability**
- **Describe patterns in primary production over space and time**
- **Compare and contrast primary production in aquatic and terrestrial ecosystems**

INTRODUCTION

Ecological questions require quantitative data. How researchers gather those data vary from project to project, but one of the important aspects of ecological research is figuring out how to quantify what is happening ecologically. **Primary production** is the conversion of solar energy into chemical energy stored in the bonds of reduced sugars and represents the base of the energy pyramid in an ecosystem. Facilitated by photosynthetic organisms, primary production can be quantified by measuring the accumulation of photosynthetic biomass over a period of time.

For this activity you will be exploring data on end of season biomass harvested from plots from the nearby field research station at Cedar Creek (and around the globe). Biomass measurements give an indication of the net primary productivity of an area (the amount of carbon fixed minus the amount of carbon used in respiration). The primary productivity of an ecosystem is dependent on the availability of factors that limit plant growth. Limiting factors for productivity

can include sunlight, water, and nutrients. At the Cedar Creek station and at other sites around the world there are experiments set up to understand how nutrient input affects grassland ecosystems. The researchers conducting these experiments have joined together into a Nutrient Network or NutNet. You can read more about NutNet here (https://nutnet.umn.edu/). In short, this global experimental network it observing the effects of nutrient addition and herbivores on grasslands.

PART 1: GRAPHING DATA & USING A T-TEST TO COMPARE BIOMASS IN FERTILIZED & CONTROL PLOTS

Graphing baseline data:

Using a t-test:

In the initial year of a field experiment, it is important to verify that you have standard starting conditions across your field sites. In order to examine the impact of fertilizers on grassland productivity, we must first establish that all biomass production at each of the study plots is the same. To do this, we can use a statistical procedure called a t-test. The t-test allows us to compare two groups and determine if there is a statistically significant difference in the mean values of the two groups. To do this, we will use the data collected by students in fall 2018 from year zero in the Nutrient Network Education (NutNEd) plots to compare the living biomass measured in control plots and fertilized plots.

Procedure:

1. Open the data file labeled "Nut Net Bootcamp Data Set 0" this is the biomass data for the NutNEd plots at Cedar Creek that was collected by students in the Fall 2018 Foundations Lab.
    a. When you open the JMP program, choose File from the taskbar and then select Open.
    b. Navigate to the data file saved on your computer and click open, this should open the Excel Import Wizard.
    c. Leave all of the settings in the import wizard at their default values and click import. This will open your file as a JMP table. Your table should contain 4 columns: "site_name, year_trt, trt, and live_mass"
2. To make things a bit easier for you, we've already subsetted this dataset so that it only includes the biomass measurements for the control plots and NPK plots from year zero in NutNEd. We will perform the t-test to see if there are any differences between the control and treatment plots in our year zero samples. A t-test is a statistical test used to test for differences in the means of two groups. In this case, we are looking for evidence that there is **no difference** between our two groups.
    a. Under the analyze menu, select the fit Y by X option. This will launch the fit Y by X window.
    b. Select the trt variable as your X factor and the live_mass as your Y response variable. Click okay to run the analysis. This will open a window containing a plot of your data.

c. In the upper right-hand corner of your plot window, there is a small red triangle that allows you to run various statistical tests. Click the arrow and select t-test from the dropdown menu.
d. The t-test analysis table should open below your plot. There are several things listed in this table, but the thing that we are looking for is called the p value. This value is located next to the label that says “Prob<t”. It is common practice for scientists to use a p value of 0.05 as their cutoff value for determining statistical significance. In other words, if your p value of a t test is greater than 0.05 there is not enough evidence for you to conclude that there is a true difference between your two groups (i.e. that any observed difference could be due to chance). Look at the p value listed in your table. What conclusion would you draw about the difference in live biomass between the control and treatment plots in year zero?

PART 2: GRAPHING PRIMARY PRODUCTION ACROSS TIME AND SPACE

Graphing Primary Production Across Time:

For the first part of this lab, we will explore how primary production changes over time in response to a nutrient addition experiment. You have been provided data from the Nutrient Network, a global network of ecologists performing standardized experiments to better understand how fertilization impacts diversity and productivity in grassland ecosystems. Using this dataset, you will use a simple linear model to answer the following questions: 1) How has primary production changed over time in grassland plots that have been fertilized with NPK 2) How does this pattern compare to what happened in the control plots over time?

Procedure:

Using the data from the Cedar Creek NutNet plots, you will work with a partner to graph the annual primary production values for each year of the experiment in both the control plots and the fully fertilized plots. You will use a simple linear regression to determine if productivity in these two plots has increased over time and compare the rate of increase in the fertilized plots to the rate of increase in the control plots.

1. Open the data file in JMP. The data file is posted on the Canvas page titled “Nut Net Bootcamp Dataset 1”
   a. When you open the JMP program, choose File from the taskbar and then select Open.
   b. Navigate to the data file saved on your computer and click open, this should open the Excel Import Wizard.
   c. Leave all of the settings in the import wizard at their default values and click import. This will open your file as a JMP table. Your table should contain 4 columns: “site_name, year_trt, trt, and live_mass”
2. Graph the live mass (ANPP) vs treatment year for control and NPK plots.
   a. Navigate to the graph tab on the toolbar and open the graph builder
   b. In the graph builder window, drag the live_mass variable to the y-axis of your graph
   c. Next, drag the year_trt variable to your x-axis

d. Finally, you want separate regression lines for the control and NPK plots. To achieve this, drag the trt variable into the overlay box. This should separate your graph into red dots and blue dots representing the two treatment types.
e. Next, you will need to change the graph type to linear regression by selecting the straight line on a scatter plot icon at the top of the graph builder window. Once you have changed the graph type, you can click done in the graph builder window.
f. In this window, you can click on the legend, axes, and other aspects of the graph to customize the visualization. Modify your figure by providing appropriate axis labels, legend labels, etc. Once you have a figure you are happy with, save your figure by going to file, export, and exporting the figure as an image file. **You will upload this image file a part of your post-lab assignment.**

3. For the last step in this activity, we will use the analyze tab to test the significance of slopes for each treatment group. In other words, we want to determine if there is statistical evidence that the slopes of our two lines are significantly different from zero (flat). Then we will compare our slope parameters to determine if our fertilized plots have a larger slope value than the control plots (a faster rate of biomass growth over time). There is a statistical test to determine is this difference in slopes is significant called an ANCOVA (Analysis of Covariance), but this test is beyond the scope of this activity.
   a. Start by opening the Fit Y by X program under the Analyze tab in the toolbar. This will open the Y by X model window.
   b. Add year_trt as the x factor and live_mass as the y response. This will result in a simple linear model relating these two variables. We want to see if there is a difference between the control plots and treatments, so add the trt variable to the By option. Hit okay to run the analysis.
   c. After you hit okay, the Bivariate relationship window will be opened. This should contain two scatter plots, one for the control plots and one for the NPK plots.
   d. In the upper left corner of the bivariate plots, you should see a small red triangle. Click the triangle to launch the fit options. Click the fit line option to produce the best first line.
   e. The best fit line should now be shown on the bivariate plot and the linear fit window will appear under the plot. This window contains a lot of information, but for our purposes we are interested in the Analysis of Variance tab and the Parameter Estimates tab.
   f. The Analysis of Variance tab tells you if the slope of your line is significantly different from zero. In the bottom right corner, there is a section labeled Prob>F, this is referred to as the P value for the statistical test. If the P value is less than 0.05, it means that there is statistical evidence that the slope of your line is not zero. Record the P value for your control plots. How would you interpret your P value?
   g. Under the parameter estimates, you will see the slope and intercept estimates for the simple linear model describing your best fit line. The slope estimate is found in the year_trt row of the table. Record the estimate for your slope parameter. How would you interpret your slope parameter?
   h. Repeat the above steps on the bivariate plot window for the NPK plots to find the P value and slope estimates for the NPK plots.

   i. Now compare the slope estimates and P values from the two treatment groups. What can you conclude about the effect of the NPK treatment on biomass production?

Graphing Primary Production Across Space:

In addition to understanding how productivity changes over time, the Nutrient Network experiment allows us to study how fertilization impacts primary productivity at different grassland sites across the planet.

Procedure:

To start, we will compare the live biomass in fertilized plots and control plots at the original Nutrient Network sites that have now been running for 10 years. Then we will explore how this fertilization effect varies by latitude.

1. Open the data file in JMP. The data file is posted on the Canvas page titled "Nut Net Bootcamp Dataset 2"
   a. When you open the JMP program, choose File from the taskbar and then select Open.
   b. Navigate to the data file saved on your computer and click open, this should open the Excel Import Wizard.
   c. Leave all of the settings in the import wizard at their default values and click import. This will open your file as a JMP table. Your table should contain 5 columns: "site_name, latitude, year_trt, trt, and live_mass"
2. Graph live biomass in control plots and fertilized plots for by study site.
   a. Open the graph builder tool. Drag the site_name variable to the X axis and the live_mass to the Y axis.
   b. To show the difference between the control and treatment plots, drag the trt variable to the overlay box.
   c. This figure can be displayed as a bar graph or a box and whisker plot. These options are found on the icon bar above the plot. Experiment with the two types of plots and choose the plot that you feel is the best way to represent this data.
   d. Once you have picked your plot type, click done and label your axes to finish your graph. **You will upload this figure on Canvas as part of your post-lab assignment.**
3. Observe the variability in the difference between fertilized plots and control plots at different study sites. What patterns do you see?
4. Calculate fertilization effect by dividing the mean live biomass in the fertilized plots by the live biomass in the control plots for each of the sites.
   a. To perform this calculation in JMP, you need to use the tables menu.
   b. Under the table menu, choose summary. This will load the summary table builder window. We want the mean live mass number for each study site, so select the live_mass variable and select mean from the Statistics drop down.
   c. Next select the site_name variable and hit the group button. This will give you the mean live_mass number for each site in a new table, but we also need to include our treatment types. Select the trt variable and hit the subgroup bottom.

d. Finally, we want our new data table to include the latitude value for each site. There isn't a great way to accomplish this using the summary table feature, so we are going to use a work around. Since the latitude value is the same for all rows of the same study site, we can simply include the mean latitude in our data table to allow us to use it for graphing. To do this, select latitude and under the statistics drop down, select mean.
e. Hit OK. This should produce a new data table that has 6 columns.
f. Finally, we need to calculate the fertilization effect for each site. To do this, create a new column at the end of your data table by double clicking in the empty column. It should create a column titled "column 7" that has no data in it. Double click on the column 7 header to open the column options window.
g. At the bottom of the window you should see the column properties drop down. Click on the drop down to reveal the column properties and select "formula"
h. In the formula window, click the edit formula button. This will open the formula editor.
i. In the formula box, use the panel on the left and the operators at the top of the window to enter a formula that divides the live mass in the treatment plots by the live mass in the control plots. Click OK.
j. In the column name field, change the name to Fertilization Effect. Click okay.

5. Plot your calculated fertilization effect against latitude and describe the relationship.
    a. With your new data table selected, open the graph builder window.
    b. Using the same procedure as you did in the previous activity, create a scatter plot with a regression line showing the fertilization effect by latitude. **You will upload this figure as part of your post-lab assignment.**
    c. What pattern does this figure show? How would you interpret this pattern?

Comparing the Impact of Different Fertilizers:

Procedure:

Using the data from multiple NutNet plots, you will work with a partner to graph the annual primary production values for each year of the experiment in both the control plots and the fertilized plots. You will use a simple linear regression to determine if productivity in these two plots has increased over time and compare the rate of increase in the different fertilized plots to the rate of increase in the control plots.

1. Open the data file in JMP. The data file is posted on the Canvas page titled "Nut Net Bootcamp Dataset 3"
    a. When you open the JMP program, choose File from the taskbar and then select Open.
    b. Navigate to the data file saved on your computer and click open, this should open the Excel Import Wizard.
    c. Leave all of the settings in the import wizard at their default values and click import. This will open your file as a JMP table. Your table should contain 4 columns: "site_name, year_trt, trt, and live_mass"

    d. Make sure to double check that your live_mass data column is formatted as a continuous numeric variable. You can double check this by double clicking on the column header to check the column properties.

2. Graph the live mass (ANPP) vs treatment year for all treatment types.
    a. Navigate to the graph tab on the toolbar and open the graph builder
    b. In the graph builder window, drag the live_mass variable to the y-axis of your graph
    c. Next, drag the year_trt variable to your x-axis
    d. Additionally, you want separate regression lines for each treatment type. To achieve this, drag the trt variable into the overlay box. This should separate your graph into different colors based on the treatment type.
    e. Finally, we want to determine if the relationship for each treatment type is the same for each of the sites we are looking at. To do this, you can use the "wrap" function in JMP. Drag the site_name variable in to the "wrap" box that is in the top right corner of the graphing window (just to the left of the overlay box).
    f. Next, you will need to change the graph type to linear regression by selecting the straight line on a scatter plot icon at the top of the graph builder window. Once you have changed the graph type, you can click done in the graph builder window.
        i. Compare and contrast the regression lines from each of the different plots. Does it appear the nutrient treatments had the same effect at each site? Would you feel confident saying there was a single nutrient (N, P, or K) that limited primary production at all sites?
    g. In this window, you can click on the legend, axes, and other aspects of the graph to customize the visualization. Modify your figure by providing appropriate axis labels, legend labels, etc. Once you have a figure you are happy with, save your figure by going to file, export, and exporting the figure as an image. **You will upload this figure as part of your post-lab assignment.**

3. For the last step in this activity, we will use the analyze tab to test the significance of slopes for each treatment group. In other words, we want to determine if there is statistical evidence that the slopes of our lines are significantly different from zero (flat). Then we will compare our slope parameters to determine which fertilized plots has the largest slope value compared to the control plots (a faster rate of biomass growth over time). There is a statistical test to determine if this difference in slopes is significant called an ANCOVA (Analysis of Covariance), but this test is beyond the scope of this activity.
    a. Start by opening the Fit Y by X program under the Analyze tab in the toolbar. This will open the Y by X model window.
    b. Add year_trt as the x factor and live_mass as the y response. This will result in a simple linear model relating these two variables. We want to see if there is a difference between the control plots and treatments as well as if those relationships were the same at each site, so add the trt and site_name variables to the By option. Hit okay to run the analysis.
    c. After you hit okay, the Bivariate relationship window will be opened. This should contain 16 scatter plots, one for each treatment at each site.

d. In the upper left corner of the bivariate plots, you should see a small red triangle. Click the triangle to launch the fit options. Click the fit line option to produce the best first line.
e. The best fit line should now be shown on the bivariate plot and the linear fit window will appear under the plot. This window contains a lot of information, but for our purposes we are interested in the Analysis of Variance tab and the Parameter Estimates tab.
f. The Analysis of Variance tab tells you if the slope of your line is significantly different from zero. In the bottom right corner, there is a section labeled Prob>F, this is referred to as the P value for the statistical test. If the P value is less than 0.05, it means that there is statistical evidence that the slope of your line is not zero.
g. Under the parameter estimates, you will see the slope and intercept estimates for the simple linear model describing your best fit line. The slope estimate is found in the year_trt row of the table.
h. In the table below, record the slope parameters for each of the sixteen regression lines (if the P value for the lines is greater than 0.05 than you should record a zero for the slope estimate).

Site Name	Treatment Type	Slope Estimate

i. Now compare the slope estimates for each treatment type within a site. What can you conclude about the effect of the different fertilizer treatments on biomass production at each of the 4 sites?

The pre- and post-lab questions for this lab exercise are two separate Canvas assignments.

PRE-LAB QUESTIONS

1. List the first 10 words that come to mind when you think of "grassland".
2. Browse https://nutnet.umn.edu/ What anthropogenic activities have altered nitrogen and phosphorus availability? How much have their availabilities increased?
3. Give a brief description of the NutNet project **in your own words**.
4. Review figure 4 from Fay et al., 2015. What pattern is shown in this figure? What conclusions do the authors draw based on this pattern?
5. Based on the two papers you read (Elser et al. 2007 & Fay et al. 2015), how would you define nutrient limitation?

POST-LAB ASSIGNMENT

This assignment consists of two parts, which you can upload to Canvas as separate files, or combine into one Word or pdf document:

1. Upload a document that contains all of the plots you have made from this lab activity (the ones indicated as being part of the post-lab in bold font in this document).
2. Upload a document with your answers to the post-lab questions.

POST-LAB QUESTIONS

1. Without looking at your first list, do a second quick word list of 10 words that come to mind when you think of "grassland".
2. Describe the pattern you observed when you graphed primary production across time for the Cedar Creek NutNet plots. Where the fertilized plots different than the control plots? Explain your answer.
3. How did the fertilization effect of the NutNet plots vary across space (latitude)? What conclusions did you draw based on the graph you made?
4. Data analysis is an important part of doing science and as scientists we often have to choose the correct tools for performing our data analysis. In this lab, you used JMP software to do your data analysis. What benefits did JMP have compared to other software you have used (Excel, R)? What drawbacks were there?

Appendix 3: Example schedule from the second semester course

Group assignments are in **bold**.

Week	Starts (Monday)	In-Class Activities	Assignments<br>Due by class time unless otherwise specified
1	Jan 20	Introductions & Overview<br>Software downloads (JMP, R?)<br>Figures, Tables and Legends<br>Form Groups	Complete pre-semester survey<br>Review proposal and/or previous 3004 posters; bring project ideas to class<br>Statistics attitudes survey due Sunday
2	Jan 27	Progress Report 1<br>Project Plan - Go over hypotheses<br>Review Assignment 1<br>Reproducibility in research; lab notebooks	Assignment 1<br>Pechenik Chapter 3 & 4
3	Feb 3	Progress Report 2<br>Project work time - datasets<br>Review Assignment 2<br>Introduce Project Plan	Assignment 2
4	Feb 10	Progress Report 3<br>Journal Club Overview<br>Review Assignment 3	Assignment 3<br>**Project Plan** - draft version due Sunday<br>**Lab notebook** check 1 - Sunday
5	Feb 17	Progress Report 4<br>Journal Club 1 - Intro focus<br>Writing: expectations for Introductions, citing sources	Journal Club 1 Questions<br>Read about Introductions & citing sources: Pechenik p.195-200 (8th ed: p.203-9)& Chapter 5<br>**Project Plan** - final version due Sunday<br>Reflection 1 due Sunday<br>Peer evaluation 1 due Sunday
6	Feb 24	Progress Report 5<br>Review Assignment 4<br>Writing: peer reviews	Assignment 4<br>Introduction draft for peer review due Sunday<br>Poster session poll due Sunday<br>**Lab notebook** check 2 - Sunday
7	March 2	Progress Report 6<br>Journal Club 2 - Methods focus<br>Writing: expectations for Methods	Journal Club 2 Questions<br>Read about Methods: Pechenik p.152-8 (8:158-64)<br>Peer review Introduction drafts by Wednesday<br>Introduction draft for TA due Friday
	March 9	Spring Break	
8	March 16	Zoom meeting with TA<br>Online slides: expectations for posters<br>Work time: Sample Figure & Legend	Read about posters: Pechenik p.233-8 (8: 242-8) & poster guidelines<br>**Sample Figure and Legend** due Sunday<br>Methods draft for peer review due Sunday
9	March 23	Progress Report 7<br>Work time: posters	Peer review Methods drafts by Wednesday<br>Methods draft for TA due Friday

			**Lab notebook** check 3 - Sunday
10	March 30	Progress Report 8<br>Journal Club 3 - Results/Discussion<br>Writing: expectations for Results<br>Poster work time	Journal Club 3 questions<br>Read about Results: Pechenik p.61-65 (8: 64-8) + p.181-8 (8: 188-95)<br>**Poster draft** due Sunday<br>Peer evaluation 2 due Sunday
11	April 6	Writing: expectations for Discussion & Abstract; feedback on Methods<br>Feedback on poster drafts<br>Work time: poster revisions	Read about Discussions: Pechenik p.188-95 (8:196-203)<br>Results draft for peer review due Sunday<br>**Final poster** due Monday 4/13
12	April 13	Progress report 9: practice poster presentations<br>Work time: writing & peer reviews	Peer review Results drafts by Wednesday<br>Results draft for TA due Friday<br>Discussion draft for peer review due Sunday<br>Comment on practice presentations by Sunday
13	April 20	Poster presentations & reviews	**Final poster presentation** due Wednesday<br>Peer review Discussion drafts by Wednesday<br>Discussion draft for TA due Friday<br>Poster reviews due Sunday
14	April 27	Lab Skills Assignment<br>Writing: feedback on Results, Discussion & Abstract; how to revise<br>Evaluations	Read about revising writing: Pechenik Chapter 6<br>Reflection 2 due Sunday<br>Peer evaluation 3 due Sunday<br>Student writing release form due Sunday<br>**Lab notebook** check 4 - Sunday
15	May 4	No class this week	Final research paper due Wednesday<br>Final course evaluation & CBS surveys due Sunday

Note: Attendance at ONE of the poster sessions in week 13 is required. Dates and times for these sessions will be announced early in the semester. Your entire group doesn't need to attend the same poster session together. If you're unable to attend any of the sessions, please inform your instructor as soon as possible.

Appendix 4: Example linear regression assignment from second semester course

Assignment 1 Linear Regression and Correlation Testing
Linear Regression

Simple linear regression describes a continuous response variable (prediction) as a function of one or more predictor variables. Remember high school math? The following is a linear model you would have learned in high school:

$$Y = mx + b$$

In a linear model, the slope (m) describes how much of an effect x has on y and b represents the intercept value (the y value when x is zero). Let's talk about the price of a small bag of rice. If I have a linear model of rice price, y, as a function of rice quantity, x, and the slope is equal to 0.00001 (m=0. 00001), then each increase in a grain of rice would result in $0.00001 increase in the price of the bag of rice. So the linear model of the rice price would be:

$$Y = 0.00001x + b$$

The parameter b tells us how much a bag of rice would be worth if it had no rice inside, so b would the cost of the bag itself. We can imagine the bag costs $0.50, so:

$$Y = 0.00001x + 0.50$$

What if we want a model to understand what leads to weight gain? Our steps would be:

Step 1: Define a dependent variable (what are we using to measure weight gain). How about body fat percentage? That would be variable y.

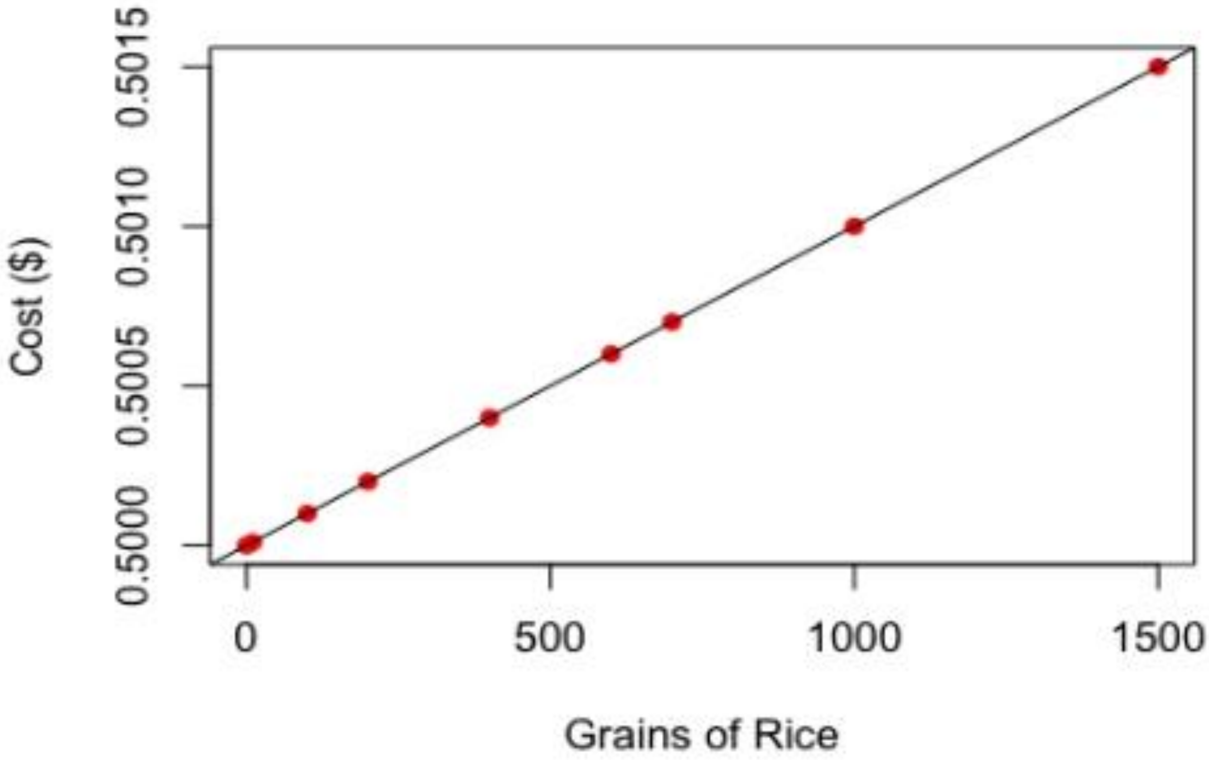

Step 2: Pick a variable that might influence weight gain. How about number of burgers eaten per week? This is the independent variable, x.

Step 3: Gather data on body fat percentage and burgers eaten per week.

Step 4: Create a regression model. In this example, body fat percentage is the dependent variable (y) and burgers eaten per week is the independent variable (x). Other influences, like starting body fat percentage, amount of vegetables eaten per week and amount of exercise per week all get combined into another parameter β that we will mention below. A plot of that data could look something like this:

Note: The data points don't fall on the line. Why? The line is a best fit that minimizes the total distance of all the data points to a straight line. It tells us that, on average, the more burgers you eat per week, the higher we can predict your body fat percentage to be. The distance from a data point to the line looks like noise or error, but it actually captures all of the other influences like exercise and vegetable intake. To include these other variables we can create a multiple variable linear equation of the form: $y = \beta_0 + \beta_1 X_1 + \beta_2 X_2 + \ldots.$

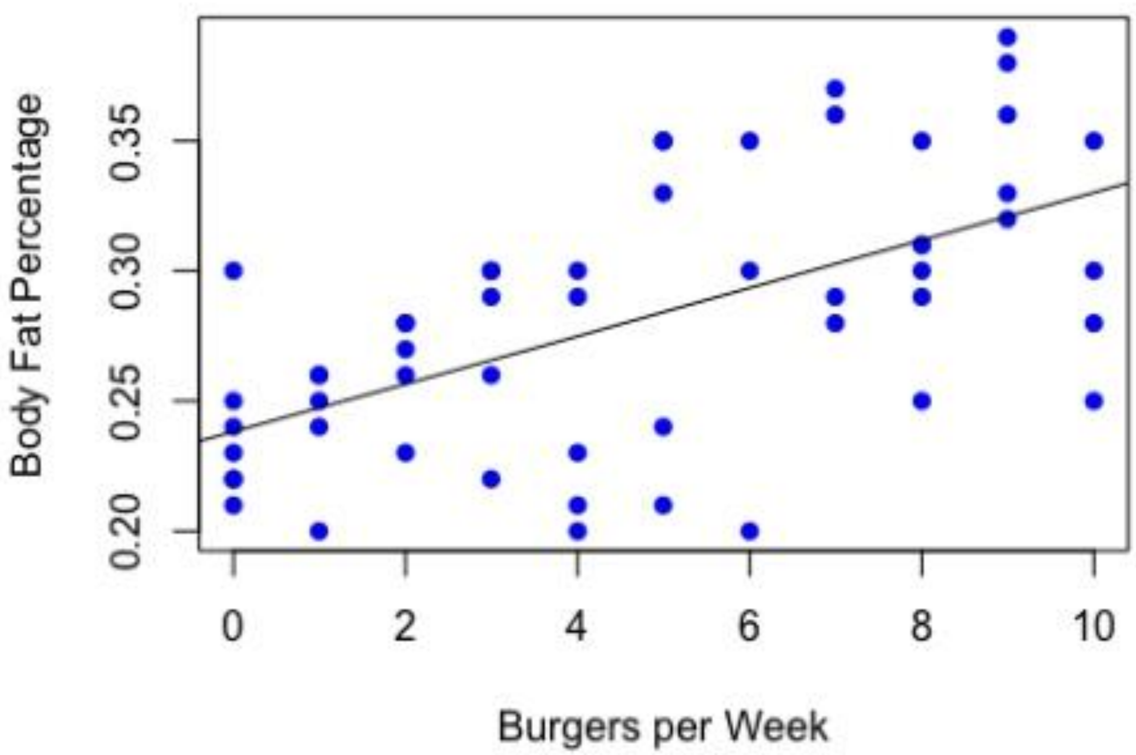

Linear Regression in JMP

In JMP, you can perform a linear regression using the analyze function. Think back to your bootcamp activity where you examined how the amount of primary production changed over time in fertilized and control plots. This was a linear regression. To build a linear model in JMP, select analyze from the menu bar and then choose “fit Y by X”. From here, you can define you x and y variables that you would like to build a regression model from.

For this assignment, you will build a linear regression model to describe the relationship between added nitrogen and aboveground productivity. You can access the data from the Long Term Ecological Data network data portal.
Click HERE.

The get the data you must:

1. Download the data file through clicking the above link
2. If the filename is e247_Aboveground Standing Crop Biomass.txt.csv, change the file name to e247_Aboveground Standing Crop Biomass.txt
3. Open the file with JMP

Using this dataset, we will build our linear regression model:
Step 1:
You will need to determine which will be your dependent variable and which will be your independent variable. The variables to choose are:

- Nitrogen fertilizer added to plot
- Aboveground biomass

Consider the experimental design of the nutrient network in making your decision.
Step 2:
Build your linear regression model using the Fit Y by X function under the analyze tab. Drag your dependent variable into the Y box and your independent variable into the X box. Hit okay to produce the scatterplot. Fit your linear regression model by clicking on the small red arrow in the top left corner of the scatterplot window and selecting “Fit Line”
From the linear fit model, record the following parameters

1. Slope (m):
2. Intercept (b):
3. R_2 value:
4. t-Ratio:
5. p-value:

In addition to the parameters above, you can find information about a number of other statistical values in this output table. Below are some short descriptions of the different values you can find (we will spend more time on this in future assignments).

Residuals tells us the variance that is not explained by the independent variable. It is the distance of the points from the best-fit line. It tells us the **Min**, **Max**, **Median** and interquartile range (**1Q**, **3Q**). If the range is roughly symmetrical (in terms of magnitude), then the data is likely normally distributed.

The **Estimate** values are the estimates of the mean of the distribution of the variable listed (intercept and slope)

The standard error (**Std. Error**) is the square root of the variance of the distribution. It is a measure of the uncertainty in the estimate.

t-value is the estimate divided by their standard errors.

Pr(>|t|) is the probability of achieving a value of t that is as large or larger, if the null hypothesis were true. Here the null hypothesis is that the estimates are individually 0 (intercept is 0 and there is no slope (the line is horizontal)).

The **significance codes** give a breakdown of what the stars (**) mean, and thus explain the p-value significance.

The **Residual standard error** tells us how much variability there is in the residuals.

The R-squared tells us information regarding the correlation between the dependent and independent variables. If the ratio is close to 1, there would be a 'perfect' correlation. If the value is 0 there is no correlation. The Adjusted R squared accounts for the number of variables in the model.

The F-Statistic tells us the significance of the entire model. It is the ratio of two variances, the variance explained by the parameters in the model (in this example we only have one) and the residual or unexplained variance. The bigger it grows, the more unlikely it is that the parameters in the model do not have any effect at all.

Appendix 5: Research paper guidelines (used in multiple research areas)

Biol 3004 Research Paper: Guidelines for Writing

Overview

In this assignment, you will be writing a scientific research paper based on your lab work this semester. Scientists write for publication in peer-reviewed journals because this is how we share our results, analyses and insights with the broader scientific community. Research papers are one of our most important products, and a project is not complete until it is published.

Writing for publication in peer-reviewed journals serves a very different function than writing a lab report. Lab reports test your understanding and ability to communicate aspects of your experiment to an instructor; generally these experiments have been repeated multiple times and have an expected result, interpretation or outcome. In contrast, a research paper aims to add to the body of knowledge within the scientific community. Research papers use data from experiments and analyses of those data to support the authors' conclusions. Other scientists who read a research paper may have their own interpretations of the data and may not agree with the authors' conclusions. They may want to repeat the experiments, or design new experiments to test alternative hypotheses that may lead to different conclusions.

Generally, research papers are not written like an essay from start to finish. Usually scientists have already performed most of the experiments, analyzed their data and drawn conclusions from the data before starting to write. Therefore, it's common to begin by creating draft versions of the expected figures and tables that will document the results obtained. The writing itself may begin with the Results section, using the assembled figures and tables (and revising them if needed during the writing). Alternatively, writing may start with the Materials and Methods (since it's often a straightforward section to write and that makes starting the writing process a little easier), or with the Introduction (to lay out the background for the study). The Discussion and Abstract are often written last, after the other sections are complete.

For this course, you'll be writing the introduction first so you have more time for project work before trying to write the other sections. Due dates for drafts are outlined on the Canvas site. For each section of the paper, you'll first submit a draft for peer review. You'll review drafts written by two other students, and receive feedback from students who read your draft. You can revise your initial draft (if you wish) before submitting it to your grad TA through a separate Turnitin assignment on Canvas. You'll receive feedback from your grad TA on each draft and have the opportunity to make revisions again before submitting the final version of your paper at the end of the semester. **The final version of your paper is due by 11:55PM on Wednesday May 6. Allow time for editing and proofreading before you submit your final revised draft!**

Purpose

This writing assignment has a number of learning objectives:

1. To give you an authentic experience of communicating experimental results in a typical format for biology.

2. To challenge you to draw specific conclusions from your data and to defend these conclusions.
3. To challenge you to integrate information from a variety of sources to construct and support your conclusions.
4. To give you the opportunity to improve your writing through revision based on critiques from your classmates and instructors.

Format & Content

Your paper should contain the sections listed below. It should be double-spaced, in 12-point font with 1-inch margins. There is no required number of pages; suggested lengths for some sections are listed below, but **these are only rough guidelines**. Previous students who've received high scores on their research papers have written at least 8 pages (not including references, figures, and figure legends). Papers more than 20 pages long should be edited to reduce their length.

In this course, project work is carried out in groups, but each student is responsible for writing their own research paper. The figures in your paper may be identical to those in the papers written by your team members, but the text should be your own. Everyone in the group is expected to contribute to making the figures for their research project.

Title page

The title should indicate what the paper is about and be interesting enough to encourage someone to read the paper. Don't forget to include your name on the title page!

Abstract (~150-250 words)

The abstract should provide a concise summary of the major aspects of the entire paper.

- Purpose of the study (hypothesis, overall question, objective)
- Description of the basic experimental design/study design
- Major findings/results
- Important conclusions or interpretations

Style

- Past tense
- Should stand on its own without reference to any other part of the paper
- Limit background information to a sentence or two, at most.
- Must be consistent with what is reported in the rest of the paper

Introduction (~2 pages, double-spaced)

- *The introduction should provide context for the work, the objectives, and the hypothesis/es.*
- What is the broad question that motivated this study? Why is the problem interesting? Establish the context for the work by summarizing our current understanding of the problem being investigated, with references to the relevant primary research literature, so the reader can understand how the study relates to previously published work.

- State the purpose of the work in the form of the hypothesis, problem, or specific question(s) it addresses. How does the work fill a gap in our understanding of this area of biology?
- Briefly explain the approach to answering the specific question(s) and the overall strategy for the study.

Style

- Use the active voice as much as possible. Some use of first person is acceptable.
- Cite primary scientific literature (see Literature Cited below) to document any information that is not common knowledge.

Materials and Methods (2-3 pages, double-spaced)

The Materials and Methods should document the necessary materials and the experimental procedures in sufficient detail that an experienced scientist could reproduce the work.

- What strains or biological starting materials were used for the experiments? How were they grown or maintained?
- What other materials did you use? Provide sources for important or unique reagents that were critical for the work.
- Describe the procedures for the study, including how data were collected (eg: numbers of cultures or time points, concentrations of reagents, number of replicates if appropriate). Reference literature sources (**not** LabArchives) for details when possible.
- Describe how the data were summarized and analyzed. How were the images or graphs in your figures obtained or created? Indicate what types of descriptive statistics were used and what hypothesis tests were performed when appropriate.

Style

- Usually written in 3rd person, passive voice. Use the past tense.
- Divide this section into logical subsections devoted to specific procedures or groups of procedures; each subsection should have its own sub-heading.
- Write in paragraphs and full sentences, not a list of bullet points.
- Cite earlier papers or manufacturer's instructions for procedures that have been published previously. If you cite a previous publication you don't need to report details of the procedure, **but you should describe any differences** between your procedure and the published one.

Results (1-2 pages, double-spaced, not including tables and figures)

The purpose of the Results is to present and illustrate the research findings.

- Present results in an orderly and logical sequence so the reader can follow the scientific "story". The order in which results are presented doesn't need to match the order in which they were obtained.
- Use **both text and figures/tables** to present your results. Data generally should be shown in figures or tables, though some may be presented only within the text.
- In the Results text, briefly provide context for the data by describing the question addressed or the approach taken. Avoid redundancy by omitting procedural details - leave them for the Materials and Methods or the figure legends. Link elements of the

story to one another with explicit logical connections between paragraphs and subsections.
- Describe important trends or point out key observations in the data shown in each figure or table. Descriptions may include objective first-order interpretation or conclusion(s) from the data, but more extensive interpretation should be saved for the Discussion. Reference each figure or table as you describe it ("Fig. 1", "Fig. 2", "Table 1", "Fig. 3", etc).
- Address unsuccessful experiments appropriately. If you had a failed attempt, but were subsequently successful, you don't need to mention the failed attempt. If an experiment didn't go as expected but you learned something from the attempt, focus on what you learned and describe any limitations on the information obtained. If an experiment was unsuccessful, state that fact and describe what you did instead and/or subsequently. Don't include lengthy descriptions of the possible reasons for failure.
- Keep in mind that **it's OK to have negative results**; these should still be presented and their meaning explained.

Style
- The Results section is usually divided into subsections with sub-headings to clarify the flow of the story.
- Use the past tense to describe what you did. Use active voice as much as possible.
- Each figure and table should be sufficiently complete to stand on its own. Every figure or table must have a **legend**, or caption, describing its contents. Legends need to be sufficiently detailed that a reader can understand *exactly* what's in the figure or table without referring to the corresponding Results text. Legends should briefly describe how the data were collected as well as what is shown in the figure or table itself. Make legends single-spaced or in smaller font to distinguish them from the main text.
- Figures and tables may be integrated into the text, or they can all be placed at the end of the paper. Number each figure according to the order in which it's discussed in the text, and present them in that same order. Number tables similarly, but with their own set of numbers. If a figure consists of several parts, each part should be labeled with a different letter.
- Be specific, accurate and precise when describing your data. Relate the results from experimental samples to negative and positive controls as appropriate.

Discussion (~2 pages, double-spaced)

The goal of the Discussion is to provide an interpretation of your results and support for your conclusions, using evidence from your experiments and the results from prior studies. The Discussion should also describe the impact of the findings.
- Briefly summarize the findings in relation to the broad question posed in the Introduction. Explain whether your hypothesis is supported or rejected. Bring the paper full circle by connecting the new information back to the big picture from the beginning.
- Explain your observations as much as possible, focusing on mechanisms. If there are reasonable alternative explanations, state them and contrast them with your own interpretation.

- What specific conclusions can you draw from your experiments? Explain how your results agree or disagree with prior research and how they add to the existing body of knowledge.
- How would you move forward with this research based on the information you obtained? Provide suggestions as to what should be done next and/or describe new questions your study raises.

Style

- Refer to work done by specific individuals (including yourself) in the past tense.
- Use the active voice as much as possible. Some use of first person is acceptable.

Acknowledgements

This section recognizes those who provided assistance with the study and/or necessary materials.

- List people who contributed to the work presented in the paper, such as group members who helped with the project, mentors who provided guidance, and anyone else who made a significant contribution to the paper.

Literature Cited

This section lists the references cited in the body of the paper. This section is extremely important for giving fair credit to the work of previous researchers, presenting your findings in the context of what has already been done, and avoiding accusations of plagiarism.

- **Cite primary scientific literature** (books, journal articles, review articles) **whenever possible**. Provide **full reference information** for the sources you cited in your paper, in the style of the journal *Cell* (see below).
- **Do not** cite LabArchives in your paper. The LabArchives protocols are based on published papers or standard biological practice. For those procedures, cite the source(s) referenced in the protocol. (If you can't identify a source for a procedure, check with your TA or Dr. Kirkpatrick.)

Style

- Reference the appropriate source after the relevant statement(s) in the main body of your paper with the author's last name and the year (in parentheses), as in the journal *Cell*. (See the References section at https://www.cell.com/cell/authors#format.)
- List references alphabetically (by last name of the first author) using the citation format of the journal *Cell*, as in these examples:
    - Article in a journal: Sondheimer, N., and Lindquist, S. (2000). Rnq1: an epigenetic modifier of protein function in yeast. Mol. Cell *5*, 163–172.
    - Article on a preprint server or other repository: De Virgilio, C., Hatakeyama, R., Péli-Gulli, M.-P., Hu, Z., Jaquenoud, M., Osuna, G.M.G., Sardu, A., and Dengjel, J. (2018). Spatially distinct pools of TORC1 balance protein homeostasis. Mendeley Data, http://dx.doi.org/10.17632/m9s42s94fc.1.
    - Article in a book: King, S.M. (2003). Dynein motors: Structure, mechanochemistry and regulation. In Molecular Motors, M. Schliwa, ed. (Weinheim, Germany: Wiley-VCH Verlag GmbH), pp. 45–78.

An entire book: Cowan, W.M., Jessell, T.M., and Zipursky, S.L. (1997). Molecular and Cellular Approaches to Neural Development (New York: Oxford University Press).

- Be wary about the quality of online information (eg: .gov websites are more authoritative than .com or .org sites). If you cite a website, give the author and/or title, date of most recent update, URL and accession date.
- If you obtained information from an individual based on his or her own experiments or experience, you may cite that person as a source (eg: J. Doe, personal communication). Before citing a personal communication in paper being submitted for publication, it's customary to get permission from the person being cited. You don't need to observe that formality in this course, but you should check if the information is published before citing a person as a source.

Resources for Writing

- "A Short Guide to Writing About Biology" (9th ed. or 8th ed.) by Jan Pechenik
- Student Writing Support from the University of Minnesota Center for Writing: http://writing.umn.edu/sws/
- Citation Management Tools (information from the University of Minnesota Library): https://www.lib.umn.edu/pim/citation
- How to Write a Paper in Scientific Journal Style and Format (from the Department of Biology, Bates College: http://abacus.bates.edu/~ganderso/biology/resources/writing/HTWtoc.html
- Quoting and Paraphrasing (from the Writing Center, University of Wisconsin-Madison): https://writing.wisc.edu/handbook/assignments/quotingsources/
- How to Recognize Plagiarism (tutorial from the School of Education, Indiana University Bloomington): https://plagiarism.iu.edu/overview/index.html
- see other links on the Writing & Research Paper Information page on the Canvas site

Grading

Research papers will be evaluated using the accompanying research paper rubric. Review the specific criteria for each section and refer to them while writing and editing your paper. Final papers will be graded out of 200 points.

Participating in the peer review process will earn up to 10 points each time:

- 5 points for submitting a draft for peer review
- 5 points for providing feedback on two peer drafts

Submissions for peer review and the feedback provided to other students will be monitored for quality. We reserve the right to withhold credit for minimally completed drafts or poor-quality feedback.

The drafts of each major section (Introduction, Materials & Methods, Results, and Discussion) that you submit for grading by your grad TA will be scored out of 10 points. A draft of the Abstract is due along with the draft of the Discussion, and will earn up to 5 additional points.

Appendix 6: Research Paper Rubric

Grading Criteria	Possible Points	Strong (A range)	Satisfactory (B-C range)	Weak (D range)
(Note: Points will be multiplied by 2 to give a final score out of 200.)	100			
**Abstract & Title (10 points total)**				
Title & Name	1	Interesting & informative title	Informative title	Title unclear or uninformative
Content: purpose of the study, experimental design, major results and conclusions	5	Accurately summarizes all aspects of the study with reference to current knowledge in the field; conveys the impact of the work	Summarizes all of the expected content	Some of the expected content is missing or described inaccurately
Organization and writing	4	Very well-organized paragraph, with little unnecessary information, that can be easily understood independently from the paper	Content is provided in a logical order, though with too much or too little detail on some points; sentences are mostly clear and grammatically correct	A significant portion of the expected content is difficult to understand because of sentence-level errors or because the paragraph lacks coherence
**Introduction (20 points total)**				
Background, context	5	Coherently and accurately summarizes relevant published information so the intended audience can understand the basis of the project; demonstrates a comprehensive understanding of relevant literature	Summarizes relevant information for the intended audience to understand the basis of the project; statements are generally accurate and demonstrate a basic familiarity with relevant literature	Information given doesn't meet expected audience needs (eg: too little or too much, or irrelevant, or overly technical); some statements are inaccurate or unsupported by evidence

Goals & strategy	3	Clearly states the purpose of the work; succinctly outlines the approach to reach the goal(s)	States the purpose of the work; indicates the approach very briefly or describes the project in some detail	Statement of purpose is ambiguous or incomplete; the approach is described in far too much or too little detail
Justification for the work	5	Creates interest in the work by presenting a strong, well-reasoned explanation of how the study results will add new information to the field and enhance our current understanding	Connects the specific study goals to an unresolved question within the broad context for the work; indicates how the results of the work will be useful	Links study goals to the broader background in a cursory way, or attempts to provide a link that is poorly reasoned or inaccurate; usefulness of the results is unclear
Organization and writing	5	Paragraphs are well-connected and in logical order, starting from broad concepts and ending with specifics; no grammar errors; scientific terminology is used accurately and defined as needed	Generally organized from broad concepts to specific details; each paragraph has a clear topic; sentences are grammatically correct; scientific terminology is generally used appropriately	Information is present but logical links or purpose are difficult to discern; sentence-level errors or mis-use of scientific terminology interfere with understanding
Use of scientific literature	2	Supports statements by citing appropriate sources; in-text citations and reference list are correctly formatted	Supports most statements by citing appropriate sources; in-text citations and reference list are correctly formatted	Some statements lack literature citations, or inappropriate sources are cited; in-text citations and reference list are incomplete or poorly formatted
**Materials & Methods (20 points total)**				
Content: organisms & materials used, maintenance or growth conditions, procedures for data collection & analysis	5	Includes all relevant content for the work	Includes most of the relevant content for the work	A significant portion of the necessary content is missing

Organization and formatting	5	Logical organization with subheadings that describe each section's content; coherent paragraphs written in past tense	Logical organization with subheadings that describe each section's content; sentences well-ordered within paragraphs; uses past tense	Lacks subheadings, or content is sometimes hard to follow because of weak organization; presents information in the form of a list
Level of detail, documentation of methods	5	Concisely describes all methods with an appropriate level of detail and literature citations, so that an experienced researcher could replicate the experiments	Describes methods with a limited amount of unnecessary detail, or occasionally omits information needed to replicate the work; description includes appropriate literature citations	Includes far too much detail, or omits information necessary to repeat the work, and/or fails to reference sources for methods
Accuracy and clarity	5	Fully accurate descriptions of procedures that could be understood by a wide range of biologists	Generally clear and accurate descriptions of procedures, but readers might be left with questions about certain aspects of the work	Procedures are hard to follow or descriptions include multiple errors
**Results (20 points total)**				
Figure format and content	4	Data are presented in an appropriate format (whether as a figure, a table or in-text); visuals are labeled unambiguously and are easy to read or interpret; analyses are thorough, appropriate, and free of errors	Generally appropriate choices of presention format (whether as a figure, a table or in-text); visuals are well-labeled so reader can understand what is shown; analyses do not contain errors	One or more figures or tables are misleading or difficult to interpret due to poor choice of format or lack of clear labeling; analyses are incomplete or contain errors
Figure legends	4	Each figure or table can be fully understood without reference to the text; titles and	Figures and tables have descriptive titles and legends, and usually can be	One or more figures or tables lack a title or legend; legends

		legends support the visuals without directly duplicating information elsewhere in the paper	understood without referring to the text	are present but incomplete or difficult to understand
Links between figures & text	2	All figures and tables are cited explicitly in the text and presented in the same order that they're cited	All figures and tables are cited explicitly in the text and presented in the same order that they're cited	Some figures or tables are not cited in the text of the Results, or are presented out of order in relation to the text
Description of results	5	Text describes all results and analyses precisely and accurately, with limited interpretation and with reference to controls as needed; text highlights important features of the data that support the overall conclusions, while acknowledging any limitations or ambiguities in the data	Each figure is described in the text; the text is consistent with the data shown in the figures and tables, and points out some features of the data that support the conclusions, but may not acknowledge ambiguity in the data	Some figures are not described in the text, or are described inaccurately; logical links between the data and the conclusions are poorly articulated, or some conclusions given in the paper are not supported by the data
Organization and writing	5	Text presents a logical sequence of evidence, with subheadings that describe each section's content; paragraphs give rationale and approach followed by findings and limitations of the data or analyses	Text presents results in an overall logical order, with subheadings that describe each section's content; text includes some context for the results presented in each figure	Lacks subheadings, or the flow of the experiments is difficult to follow because of weak organization or lack of rationale for each experimental step
**Discussion (20 points total)**				
Conclusions	5	Provides insightful interpretation and mechanistic explanation of the data presented in the Results;	States reasonable conclusions based on further interpretation or evaluation of the data presented in the	Conclusions include logical errors or are only tangentially related to the data and analyses presented in the

		thoughtfully addresses limitations of the data and offers reasonable alternative interpretations for the results when possible	Results; identifies some limitations of the study; offers a biological explanation for the results if possible	Results; limitations of the data and possible biological explanations for the results are not considered
Significance of the work	5	Thoughtfully evaluates the data in relation to relevant published results; relates the conclusions to the broad context for the work as described in the Introduction; describes how the study adds to the field	Describes the potential significance of the results obtained; points out connections between the new information and the broad context for the work as described in the Introduction	Gives only a cursory treatment of the possible impact of the study, or fails to connect the conclusions to the broad context for the work as described in the Introduction
Future directions	3	Succinctly describes the most logical next step(s) for follow-up work that would add to the existing body of knowledge	Briefly outlines future experiments that could follow from the results obtained, or new questions raised by the study	Fails to consider future directions for the work
Organization and writing	5	Paragraphs are well-connected and in logical order; no grammar errors; take-home message(s) stated overtly in language that can be understood by a general audience	Generally well-organized; includes some take-home message(s); each paragraph has a clear topic; sentences are grammatically correct	Logical links or purpose are difficult to discern; sentence-level errors or disorganization within paragraphs interferes with understanding
Use of scientific literature	2	Conclusions and significance of the work are described with reference to the scientific literature; statements are supported by citing appropriate sources	Supports most statements by citing appropriate sources; demonstrates some familiarity with relevant literature	Some statements lack literature citations, or inappropriate sources are cited; demonstrates little familiarity with relevant literature

**General quality of writing (10 points)**	10	Writing is concise, direct & unambiguous; scientific terminology is used correctly; writing is free of grammatical errors and can be understood by a broad biological sciences audience; overall organization is logical and follows conventions of scientific research papers; consistently cites high-quality sources (primarily peer-reviewed literature) to support statements; in-text citations & reference list are correctly formatted	Writing is mostly direct and follows conventions of standard English; scientific terminology is used correctly; writing is targetted for the expected audience but may sometimes be difficult to follow; overall organization differs from scientific literature conventions in only minor ways; mostly cites peer-reviewed literature or other authoritative sources for statements that are not common knowledge; in-text citations & reference list are correctly formatted	Writing is overly wordy or ambiguous, contains many grammatical errors, or misuses scientific terminology; writing assumes extensive scientific knowledge, or a completely non-scientific audience; text is disorganized and/or deviates substantially from scientific literature conventions; cites very few sources, or non-peer-reviewed sources, to support statements; in-text citations and reference list are incomplete or poorly formatted

Appendix 7: Poster guidelines (including rubrics)

BIOL 3004 Poster Guidelines

Overview

The purpose of these posters is to communicate the results of your independent research projects to your fellow BIOL 3004 students, to BIOL 1961 students, and to other interested scientists from outside the course. Poster sessions will be held from April 20-24. Making the poster is a group assignment. Every group member is expected to attend one of the poster sessions to present their group's poster, either alone or together with other members of their group. In addition, each student is expected to review at least three posters produced by other groups in the course.

Schedule

- Submit a draft poster (PDF file) to Canvas by 11:55PM on Sunday April 5
- Receive feedback in class and/or in writing during the week of April 6
- Submit final poster (PDF file) for printing by 11:55PM on Monday April 13; submit the same file to the "Final poster" assignment on Canvas for grading by your grad TA
- Pick up your printed poster when notified that it's ready (during the week of April 13)
- Attend one (or more) of the scheduled poster sessions. The times and places are:
- Monday April 20, 3:45-5:30PM in 114 Bruininks
- Wednesday April 22, 3:45-5:30PM in 114 Bruininks
- Thursday April 23, 4:45-6:30PM in 64 BioSci
- Friday April 24, 1:45-3:30PM in 105 Cargill

Bring your poster with you to the session if you're scheduled to present during that time.

- Submit poster reviews online by 11:55PM on Sunday April 26

Poster format

The maximum final poster size is **36 inches high x 44 inches wide** (in landscape orientation). It's probably easiest to make your poster as a single large Powerpoint slide, but you can use any software that all members of your group are comfortable using. The final files you submit for printing and to Canvas **must be in PDF format**.

There is lots of advice about making effective posters available online; some links and recommendations for format and design are given below. If desired, you can download a poster template as a starting point for your poster. If you do, be sure to adjust the dimensions of the poster to the size above before starting to work on your design.

Posters are a visual medium for communicating your work to potentially interested people, with an opportunity for back-and-forth interaction between you and your audience. Ideally, posters should be viewable from a distance to give people an idea of what your work is about and what you've found, in a way that encourages them to come closer and talk with you to learn more about it. At some scientific meetings posters are displayed continuously, even when the author isn't present; thus it's also useful to design posters so a viewer who doesn't have the chance to talk with you can look at your results and understand your project.

Some additional resources for information about poster design:

- Chapter 11 of "A Short Guide to Writing About Biology" (by J. Pechenik)

- p. 233-238 of the 9th edition; p. 242-248 of the 8th edition
- from the U of M Libraries: Communicating Your Research with Posters
- Designing Conference Posters (by C. Purrington): long version & short version
- NeuroWire blog: How to make your scientific posters stand out

Recommendations:
- Limit the amount of text on your poster to ~600 words or less. Use visuals (with informative labels) to convey your message when you can. Use bullet points rather than paragraphs, though short paragraphs may be effective in some situations.
- Use large font sizes for text, including labels on figures. To ensure your poster can be read from a distance, print out a test version on an 8.5x11 sheet of paper and check that you can read all the text. Pay special attention to axis labels on graphs and to the text in tables and legends: **all** text should be legible from a distance.
- Show your test print to a friend to check the overall flow and visual appeal of the poster.

Specific tips from the University Imaging Centers (where your poster will be printed):
- Fonts: If you submit a file with a non-system font (meaning one you downloaded from the internet), you must either supply us with the font, or convert your file to PDF.
- Certain effects in PowerPoint, such as transparencies and gradients, do not translate properly to print. If you are using such effects we recommend printing a small proof for review. [Note: Printing a small version on your own printer may give you an idea about whether a specific effect will work or not, but results with the UIC printer may be different. It's best to avoid these effects rather than have your final poster turn out looking odd.]
- When inserting charts, figures, and photos into your PowerPoint file, please use either the "Insert" menu, or the "Paste Special" command. Do not do a simple cut and paste, otherwise there may be printing errors.
- If you're working with an Illustrator or InDesign file, please make sure all images are embedded, and that your text has been converted to outlines. [Do this before making a PDF, then check that the PDF looks OK before you submit it.]
- Prints will have a 1/4 inch white border outside of the printable area.
- Make sure that there is at least once inch of space between the page border and any text, photos, or other objects.

Poster content
Your poster should contain the sections listed below. You can include other elements as needed to describe your work. Your poster does **not** need to cover every aspect of your project: pick the key results (or a single result) and focus on communicating those key points effectively. You can elaborate further on your work when you present the poster, if some people want to hear more details or additional results.

Title of project, names of authors
Introduction or Background should include:
- statement of the general problem or question
- brief, relevant background information to show how the project adds to prior knowledge
- hypothesis

- literature citations
Methods should include:
- outline of experimental design or analysis strategy
- diagrams or flowcharts are often helpful
- what was measured & how it was measured, with a brief rationale for any procedures that might not be obvious

Results should include:
- statements about what worked (or not), and the main findings or results
- figures, graphs, images and/or tables presenting results
- occasionally data may be presented as text
- appropriate labels and figure or table legends (captions) so results can be readily understood without a verbal explanation
- if multiple results are shown, there should be a logical flow or order in the presentation

Conclusions should include:
- statement of overall conclusions (which can include whether the hypothesis was supported by the data analyzed)
- relationship between the findings and previously available information
- future directions based on results obtained, including recommended changes in approach or procedures if necessary

Literature cited or References

When thinking about poster content and format, it's often helpful to look at examples - both good and bad examples can serve as sources of ideas about what to do (or not do). Check out the posters all around the basement level of BioSci, or look at examples online, and discuss them with your group members as you're planning your own poster design.

Poster printing
Upload your final poster in **PDF** format to the University Imaging Centers printing site (http://uic.umn.edu/node/add/uic-poster-submission) by the submission deadline. Your file must not be larger than 20MB. In the Contact section, enter **your own contact information**, with **Catherine Kirkpatrick** in the PI Name spot, **Biol 3004** as the class, and **CBS** as the college. Enter your group number, including a letter or two to indicate your research area: C for computational microbiology, E for microbial experimental evolution, GE for global change ecology, M for zebrafish microbiome, or Z for zebrafish environmental toxicology. You may choose to pick up your poster in 23 Snyder Hall (St Paul), 1-151 Jackson Hall, or 1-220 Cancer & Cardiovascular Research Building (both Minneapolis East Bank).

Poster sessions & poster reviews
Students are responsible for bringing their group's poster to each session where a group member is scheduled to present it.

Each poster session will be divided into two halves of about 45 minutes each. Unless multiple people from the same group are presenting at the same session, each poster typically will be presented in either the first or second half of a session. Poster sessions will be a bit longer than 1.5 hours to allow some time for taking down posters at the end of the first half and putting up the posters for the second half.

Every student will be expected to review at least three posters produced by other groups in the course. One of these posters will be assigned to you (to ensure that every group gets a minimum number of reviews); for your other reviews you can pick any posters you wish. You'll need to listen to a presentation about each poster you're reviewing so you can evaluate the presentation as well as the poster itself. Generally students will be assigned to evaluate a poster in the other half of the same session where they're scheduled to present, and most students complete their other reviews during the same session. You're welcome to attend other poster sessions to complete additional reviews if you wish.

Reviews will be submitted through an online form, and are due by 11:55PM on Sunday April 26. Printed copies of the questions will be available during the regular poster sessions for those who want to take notes about a poster and submit their review later, but paper reviews will not be accepted for credit. You'll earn 8 points for each of your 3 reviews, for a total of 24 points. You can earn extra credit by attending an additional poster session (other than the one at which you are presenting) and submitting up to two additional reviews (for 2 points each).

Grading

In addition to submitting your final poster PDF for printing, you must also submit it through the "Final poster" assignment dropbox on Canvas. Your grad TA will evaluate your poster for completeness using the criteria under Poster content above, as well as for overall effectiveness and organization. Posters will receive a final grade out of **100 points** based on **75 points** from your grad TA and **25 points** from poster reviews (see below).

Poster grading by grad TAs: 75 points

Framing the research question, approach & methodology: 20 points

**Criteria**		**Strong**	**Satisfactory**	**Weak**
**Introduction/ background 10 pts.**	Does the poster relate the research project to an important issue or a gap in knowledge?	Poster makes the importance of the research question clear by providing background context for the work (with citations) and describing how the work addresses a knowledge gap. 9-10 pts	Poster states a research question and/or hypothesis, connects them to a relevant background or context, and suggests how the results of the work will be interesting or useful. 7-8 pts.	A research question or hypothesis is hard to identify, or very little background is provided. The utility of the results or value of the project is unclear. 0-6 pts.

**Approach & methodology 10 pts**	Is it easy to understand how the data were gathered and how the analyses were performed?	The steps of the project (data collection and/or analysis) are well explained or diagrammed. 9-10 pts.	Descriptions of the study design and methodology are mostly clear and accurate, but viewers might have questions about some aspect of the work. 7-8 pts.	Descriptions of the approach, methodology and/or analysis are missing or hard to understand. 0-6 pts.

Project results & analysis: 40 points

Criteria		Strong	Satisfactory	Weak
**Data figures and tables 10 pts.**	Are data graphed or presented in an appropriate format? Are the data figures easy to interpret?	Figures, tables and legends are very easy to read and interpret; they meet the "Acid Test" criteria of the Sample Figure & Legend assignment. 9-10 pts.	Generally appropriate choices of presentation format, but some data may be graphed in a format that doesn't support the intended interpretation. Visuals are labeled well enough to read easily and have explanatory legends. 7-8 pts.	Some figures are difficult to interpret due to defects in labeling and/or legends, or data presentation is significantly flawed or misleading. Labeling and legends are insufficient for the reader to understand the results. 0-6 pts.
**Use of figures, images and diagrams 10 pts.**	Are the visuals useful and relevant to the overall message of the poster?	Appropriate number and types of graphics are used to convey the work and support the take-home message. Visuals are attractive, large enough to see details, and easy to understand. 9-10 pts.	One or two of the visuals are not relevant, not large enough, or hard to understand, but the poster take-home message is still apparent. 7-8 pts.	Insufficient use of visuals diminishes the impact of the poster, or the visuals distract from, rather than helping to convey, the overall message. 0-6 pts.

**Results and conclusions 10 pts**	Does the poster clearly state the main findings of the study? Are the conclusions supported by the data?	The results and conclusions are clearly but briefly stated in text. Data analyses are free of errors. The conclusions represent a reasonable interpretation of the data. 9-10 pts.	Data analyses are fairly complete but may have minor flaws; conclusions are stated, and are mostly consistent with the data shown. 7-8 pts.	Data are presented in figures with no statement of results or conclusions, or data analysis is flawed so that some conclusions are not supported by the data shown. 0-6 pts.
**Context for findings & follow-up work 10 pts.**	Does the poster relate the findings to what was already known and suggest appropriate follow-up work?	Poster explicitly relates conclusions to prior knowledge in the field and makes insightful suggestions for follow-up work. 9-10 pts	Results and conclusions are stated, though their relationship to prior knowledge may not be very clear; follow-up work is described. 7-8 pts.	Results or conclusions are hard to understand, or their relationship to prior knowledge isn't described, and/ or follow-up work is missing. 0-6 pts.

Organization & layout: 15 points

**Criteria**		**Strong**	**Satisfactory**	**Weak**
**Organization & layout 10 pts.**	Is the poster well organized and easy to read, with an effective layout? Is the text sufficient to explain the graphics, but not overwhelming to the reader?	Individual parts of the poster combine to form an easily navigated unit; spacing and flow allow the viewer to follow the story readily. Graphics combine well with text (that is brief and easy to read) to deliver the message. 9-10 pts.	Individual parts of the poster are solid and the overall flow is mostly clear. Poster may have too much text, or overly small text, or the text may sometimes fail to fully explain the graphics. 7-8 pts.	Poster is crowded, poorly organized, or lacks logical flow; viewer may be confused about order of parts and where to focus. Poster contains too much text to read easily, or text and graphics don't work well together. 0-6 pts.

**Visual appeal & quality 5 pts.**	Do the chosen colors and fonts enhance the poster's impact? Is the text free of errors?	Poster is pleasing to look at; good use of color and graphics highlights the work. No spelling or grammatical errors. 5 pts.	Color scheme is appealing, though text may be hard to read in a few places due to font choice or lack of contrast. Only occasional spelling or grammatical errors. 4 pts.	Color scheme or fonts are distracting rather than adding to the effectiveness of the poster. Contains multiple spelling or grammatical errors. 0-3 pts.

Poster review questions: 25 points (average of all reviews)

Content, organization & aesthetics (15 pts)

**Organization & layout 4 pts.**	Is the poster well organized and easy to read, with an effective layout? Is the text sufficient to explain the graphics, but not overwhelming to the reader?	Individual parts of the poster combine to form an easily navigated unit; spacing and flow allow the viewer to follow the story readily. Graphics combine well with text (that is brief and easy to read) to deliver the message. 4 pts.	Individual parts of the poster are solid and the overall flow is mostly clear. Poster may have too much text, or overly small text, or the text may sometimes fail to fully explain the graphics. 3 pts.	Poster is crowded, poorly organized, or lacks logical flow; viewer may be confused about order of parts and where to focus. Poster contains too much text to read easily, or text and graphics don't work well together. 0-2 pts.
**Framing the story, giving context to the research 3 pts.**	Does the poster relate the research project, and the conclusions from the study, to an important issue or a gap	Poster makes the importance of the research question clear by providing background context (with citations) and relating conclusions to prior knowledge in the field. 3pts	Poster states a research question and/or hypothesis, connects them to a relevant background or context, and suggests how the results of the work will be interesting or useful.	A research question or hypothesis is hard to identify, or very little background is provided. The utility of the results or value of the project is unclear. 0-1 pt.

	in knowledge?		2 pts.	
**Figures, images, diagrams 3 pts.**	Are the visuals useful and relevant to the overall message of the poster? Are the data figures easy to interpret?	Visuals are attractive, large enough to see details, well labeled & easy to understand. Use of graphics supports the take-home message and draws in viewers. 3 pts.	One or two of the visuals are not relevant, not large enough, or not labeled sufficiently, but the poster take-home message is still apparent. 2 pts.	The poster has very few visuals, or too many of the visuals don't help convey the overall message of the poster. 0-1 pt.
**Experimental approach, results and conclusions 3 pts**	Is it easy to understand how the data were gathered? Are the results stated clearly? Are reasonable conclusions and follow-up work specified?	The steps of the project are well diagrammed and/or explained. The results and conclusions are clearly but briefly stated in text. The conclusions seem logical based on the data, as does any proposed follow-up work. 3 pts.	The steps of the project are mostly clear. Conclusions are stated and seem mostly consistent with the data shown in the figures. Follow-up work is described. 2 pts.	The approach or methods are not described well enough to understand them. Data are presented in figures with no statement of results or conclusions. No attention is paid to follow-up work. 0-1 pt.
**Visual appeal & quality 2 pts.**	Do the chosen colors and fonts enhance the poster's impact? Is the text free of errors?	Poster is pleasing to look at; good use of color and graphics highlights the work. No spelling or grammatical errors. 2 pts.	Color scheme is appealing, though text may be hard to read in places due to font choice or lack of contrast. Only occasional spelling or grammatical errors. 1 pt.	Color scheme or fonts are distracting rather than adding to the effectiveness of the poster. Contains multiple spelling or grammatical errors. 0 pt.

Oral presentation of poster (10 pts)

**Mastery of topic; ability to answer questions 5 pts**	Did the presenter explain the project effectively?	The presenter highlighted the main points, explained the approach and results, and was able to answer questions about the work. 4-5 pts.	The presenter did a fair job explaining the project and was able to answer some questions about the work. 2-3 pts.	The presenter struggled to explain or answer questions about the work, and appeared not to understand some parts of it. 0-1 pt.
**Engaging explanation of poster 5 pts**	Did the presenter guide you through the poster in an enjoyable way?	The presenter made the work interesting and responded to their audience. The presenter spoke clearly and was easy to follow. 4-5 pts.	The presenter explained the project but didn't engage much with their audience or was somewhat hard to follow. 2-3 pts.	The presenter read the poster rather than explaining it, or they were hard to understand and/or didn't interact with their audience. 0-1 pt.

Please comment on the poster, including which features most helped you understand the research project and what was most confusing or difficult to understand.

Appendix 8: Example Student Projects

This page is intentionally left blank. Examples are below.

How Changes in CO_2 Emissions Differ Between Continuous and Discontinuous Permafrost Zones

Abstract

Permafrost has historically been a carbon sink and it may change to a carbon source. In this study our goal was to determine how arctic permafrost is changing in relation to the heating climate. There is little understanding of how the different types of permafrost are responding to climate change in relation to one another. We chose to analyze the net ecosystem exchange (NEE) data of continuous and discontinuous permafrost from 1998 to 2016 obtained from the Arctic. We hypothesized that both discontinuous and continuous permafrost zones would show positive NEE values over time. In comparison to continuous zones, discontinuous zones were predicted to release more carbon due to more plentiful ice cracks that promote thawing; also, discontinuous zones would respond to changes in the climate more dramatically due to sunlight penetration through ice cracks which warms deeper ice layers. We ran a regression on both types of permafrost zones to determine the relationship between NEE and time as well as an ANOVA on both to determine the relationship between the two. We found that continuous permafrost zones are changing more quickly, with a NEE rate that is increasing more quickly than discontinuous zones. We also found that discontinuous zones are emitting more carbon into the atmosphere than continuous zones. This indicates that continuous permafrost zones will likely begin to resemble discontinuous permafrost zones in the future and emit much more carbon into the atmosphere, potentially leading to the arctic permafrost becoming a major source of atmospheric carbon.

Introduction

The effects of global climate change have become the subject of intense public and scientific concern within the 21st century. As the climate changes the atmosphere is expected to have an average increase in heat of at least 2.7 °F over the next 70 years (Collins et al., 2013). One of the areas that has already been rapidly changing due to this heating are regions of arctic tundra. Tundra regions have already been observed to decrease by 30 centimeters a year (Walker, 2007). As this loss of permafrost in the tundra occurs a startling relationship between climate change and permafrost reveals itself. One of the main agents of global heating is the greenhouse effect caused by a variety of gases in the atmosphere. One gas, CO_2 has been the subject of interest due to its radical increase in the atmosphere due to human activities (Dlugokencky et al., 2019). With the relationship between CO_2 and climate change being apparent, a related relationship with permafrost mentioned above is also revealed. Regions of tundra have acted as large carbon sinks for at least the last 2,000 years (Billings et al., 1982). As more permafrost melts due to heating, more CO_2 is expected to be released into the atmosphere leading to further heating and potentially a runaway feedback loop of warming that could devastate the ecology of the tundra (Oechel et al., 1993).

There has been much research done on the tundra such as Romanovsky, 2001, Ma et al., 2019, and Billings et al., 1982. Numerous measurements of Gross primary production (GPP), net ecosystem production (NEP), carbon flux, organic matter content, net ecosystem exchange (NEE) and many other factors have been made throughout many regions with permafrost by the Arctic Data Center. This existing research has indicated that the melting of permafrost is increasing and with that the rate of ecosystem exchange is also changing (Segal et al., 2016). The

exchange of carbon and other greenhouse gasses such as methane in tundra has increased throughout the 20th and 21st century and is expected to continue to increase (Marrero, 2010).

One thing that is often overlooked when studying changes in regions of tundra is the fact that there exist many types or zones of permafrost, continuous, discontinuous, sporadic, and isolated (Lantuit, 2016). Each type of permafrost exhibits unique qualities that may change how they respond to the changing climate. For example, discontinuous permafrost exhibits melting from both top and bottom unlike continuous permafrost (Romanovsky, 2001) There has been previous research on how organic matter and matter densities differ between the different zones such as in Ma et al., 2019. While there is data and research on how climate warming will affect tundra and permafrost as a whole, we do not know how this heating will affect each zone of permafrost independently and how these differences are significant. Insights in to how each of the different zones will function as carbon sinks or sources and how they will interact with the future climate could be gained from research into how these zones are affected. Of the zones, continuous and discontinuous permafrost are of particular interest due to their high abundance and regular distribution throughout the arctic (Lantuit, 2016).

This study has the goal to understand how the NEE has changed in the last decade in permafrost, specifically in continuous and discontinuous permafrost zones. We utilized eight datasets from across the northern hemisphere to analyze the trends in NEE using longitudinal and temporal data. Linear regressions of each permafrost zone were plotted in order to understand the temporal relationship with NEE in each zone and then an ANOVA was conducted to analyze the relationship between the changes in the different zones. This allows us to ascertain if there is a significant difference in the effects of climate change on different zones of permafrost, what degree this difference is, and if one zone should be given priority to preserve.

We aimed to answer the question of how soil carbon exchange into the atmosphere has changed in continuous and discontinuous permafrost. We hypothesized that both discontinuous and continuous permafrost zones will show positive net ecosystem exchange values over time. In comparison to continuous zones, discontinuous zones will release more carbon due to more plentiful ice cracks that promote thawing; also, discontinuous zones will respond to changes in climate more dramatically due sunlight penetration through ice cracks which warms deeper ice layers.

Methods

Study Sites

NEE data was compiled from five different sites, three of which were in continuous permafrost zones and two of which were in discontinuous permafrost zones. The continuous permafrost sites were Thule, Greenland; Barrow, Alaska; Svalbard, Norway. The discontinuous permafrost sites were Imnavait Creek, AK; Eightmile Lake, Alaska. The data on NEE spanned a time period from 2008 to 2018 for the discontinuous sites and 1998 to 2016 for the continuous sites. The NEE data from each site did not span the entire period but they all fell within the specified range. NEE or CO_2 flux data (which is interchangeable) was retrieved from the data sets. If there was not NEE or Carbon Flux measurements, the NEE was calculated by adding ecosystem respiration to the gross primary production (Kramer et al., 2002).

The Thule, Greenland data set and the Barrow, Alaska data set contained CO_2 flux that was collected via soil probes and consisted of three different data sets (Konkel, 2017) (Oechel et al., 2001). The date format of each was standardized. The data was also subset for the control treatment type as the original data had experimental treatments that would affect the results of

our analysis. The data from Barrow was expunged of any measurements of -9999 as those were readings that were not usable. The Kangerlussuaq, Greenland site measured NEE using a custom-built clear acrylic chamber and an infrared gas analyzer (Post et al., 2016). The data was subset by treatment type in order to avoid any experimental treatments from the study in Post et al., 2016. The Eightmile Lake, AK site measured NEE using an infrared gas analyzer (Schuur, 2018). The site data was subset for NEE and the first six months of data had to be deleted due to not having readings for NEE. The date format was standardized. The Imnavait Creek, AK site contained two separate sets for two separate years (Bret-Harte et al., 2018). Both sets had measurements for GPP and R and the NEE was calculated from these measurements. The date format was changed to match the Eightmile Lake, AK format and then was added to the Eightmile Lake, AK data.

Data Analysis

The variables selected in this study were permafrost type, month, NEE, and Month x Type. NEE, measured in Micromoles of carbon per Meter squared per second, was plotted against month. The NEE was treated as the dependent variable and the date was treated as the independent variables. Data analysis was conducted in JMP software. Two linear regressions were made, one for the continuous data and one for the discontinuous data, this was to determine the trend in NEE over the last decade and to see if there was a significant relationship between date and a change in NEE. The p-value and R^2 values were recorded for the two tundra types. An ANOVA test was run against the two types of permafrost in order to determine any significance between the changes in NEE in the continuous and discontinuous sites. Figure 2 was created using the location of each site and a blank map using Adobe Photoshop software.

Results

Net ecosystem exchange in tundra is increasing (Figure 1). Both the discontinuous and continuous permafrost zones displayed positive changes in NEE between 1998 and 2016. Continuous zones exhibited a greater change in NEE than discontinuous zones. The continuous permafrost change is given by the slope: NEE= 0.005896x, with an R^2 value of 0.25 and the discontinuous zones with the slope: NEE= 0.003756x, and an R^2 value of less than 0.0001.

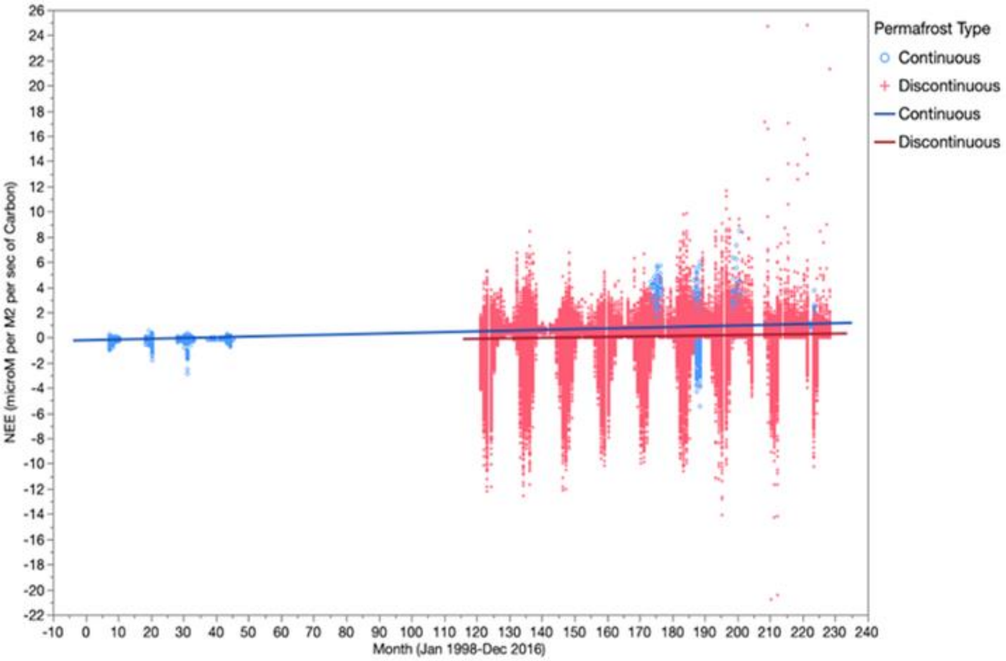

Figure 1. Net Ecosystem Exchange (NEE) of Continuous Permafrost and Discontinuous Permafrost Zones 1998-2016. There were three sites of continuous and two sites of discontinuous plotted together. The x-axis is measured in months since January 1998 with January 1998 being the 0 on the x-axis. Each dot represents a daily NEE measurement within a given month. A positive correlation was shown in both permafrost zones (Linear Regression, $R^2 = 0.003338$, $P < 0.0001$, discontinuous slope: NEE= 0.003756x - 0.5058, continuous slope: NEE= 0.005896x -0.1707).

Discontinuous permafrost zones were measured to have a greater positive NEE estimate (greater total release of carbon in the form of CO_2) than continuous zones, given by the mean values of each regression 0.145 versus 0.066, respectively.

The different rates of carbon release and the different rates in change of carbon release between continuous and discontinuous permafrost zones was also found to be statistically significant from the ANOVA test resulting in a P-value less than 0.0001.

Discussion:

Our study revealed important information about the different ways in which permafrost is changing in response to a heating climate. From the analysis that was conducted it is clear that the levels of CO_2 being emitted from regions of tundra is increasing. The positive correlation found between net ecosystem exchange and time for both continuous and discontinuous permafrost demonstrates this. The greater slope exhibited by continuous permafrost zones means a greater increase in CO_2 emissions. These results went against our original hypothesis that the discontinuous zones would show greater changes in NEE. This was highly unexpected as we believed it would be logical for the discontinuous permafrost, consisting of less dense ice coverage to show changes in NEE more quickly. This likely indicates that there are many factors that we failed to account for that would explain continuous permafrost zones changing more quickly than discontinuous permafrost zones. The increase in CO_2 emissions from permafrost zones is consistent with other studies such as Schaefer et al., 2011.

The fact that continuous zones of permafrost are responding more quickly to climate change may indicate that they will begin to resemble discontinuous permafrost zones in the near future and more ice melts and cracks form in the ice and frozen soil. This would be a drastic change as our study found that while continuous zones are changing more rapidly, discontinuous zones are emitting much more carbon into the atmosphere indicated by the larger mean value. This finding makes sense as there are more areas in the discontinuous permafrost soil and ice to allow trapped carbon to escape into the air (Pollard, 2018). This implies that if continuous

permafrost zones are going to begin to resemble discontinuous zones in the future, there will be much more carbon being released into the atmosphere from regions of tundra.

All of this demonstrates that arctic tundra is quickly becoming a source of atmospheric carbon rather than a sink. Despite this insight, there is much more research and analysis that needs to be conducted on arctic permafrost. If we are to more thoroughly understand the implications of continuous permafrost zones responding to climate change more rapidly than discontinuous permafrost zones we would want to collect data spanning further into the past and many more sites that encompass more than just the western hemisphere. We would ideally get data on how both continuous and discontinuous permafrost zones have changed in the last 50 to 100 years in order to see what type of curve the NEE change follows and determine with greater confidence what point the continuous zones will resemble the discontinuous zones. If this study was to be conducted again, care should be taken when compiling data as there were strange outliers in the continuous data sets that had to be expunged due to odd treatment groups being included with the control data.

Overall, our findings are in line with similar studies such as Biskaborn et al., 2019, which demonstrated that arctic permafrost is indeed melting and Schaefer et al., 2011, which demonstrated that CO_2 emissions are increasing from permafrost. Our study however built on the knowledge of how carbon emissions are changing differently in the different zones of permafrost. The extent of which this will affect the greenhouse effect was studied in González-Eguino et al., which found that an increase of emissions from permafrost could increase the cost to society by up to 17%. Based on our analysis and understanding, our findings are in line with the conclusion that the additional carbon emitted from permafrost zones will have an effect on the greenhouse effect which will increase as NEE also continues to increase in different

permafrost zones. Attention should be placed on the arctic permafrost as a future source of atmospheric CO_2 as it could potentially change the course of climate change.

References:

Billings, W. D., Luken, J. O., Mortensen, D. A. and Peterson, K. M. (1982). Arctic tundra: A source or sink for atmospheric carbon dioxide in a changing environment? Oecologia 53, 7–11.

Biskaborn, B.K., Smith, S.L., Noetzli, J. et al. (2019) Permafrost is warming at a global scale. Nat Commun 10, 264.

Collins, M., R. Knutti, J. M. Arblaster, J. - L. Dufresne, T. Fichefet, F. P., X. Gao, W. J. Gutowski, T. Johns, G. Krinner, M. Shongwe, C. Tebaldi, A. J. Weaver, and M. Wehner, (2013). Ch. 12 Long-term Climate Change: Projections, Commitments, and Irreversibility. In Climate Change 2013 - The Physical Science Basis (Stocker, T., Qin, D. and Plattner, G.-K.), pp.1076–1136.

Dlugokencky, E.J., Hall, B.D., Montzka, S.A., Dutton, G., Mühle, J., Elkins, J.W. (2019). Atmospheric composition. In State of the Climate in 2018, Chapter 2: Global Climate. (Bulletin of the American Meteorological Society, 100(9)), pp.48–50.

Eric Post, David Eissenstat, and Sean Cahoon. (2016). Plot net ecosystem exchange (NEE) from Kangerlussuaq (2012 - 2014 growing seasons). Arctic Data Center.

González-Eguino, M., Neumann, M.B. (2016). Significant implications of permafrost thawing for climate change control. Climatic Change 136, 381–388.

Julie McKnight Konkel. (2017). Soil CO2 flux, temperature, and moisture, Thule, Greenland 2016. Arctic Data Center.

Kramer, K., Leinonen, I., Bartelink, H.H., Berbigier, P., Borghetti, M., Bernhofer, C., Cienciala, E., Dolman, A.J., Froer, O., Gracia, C.A., Granier, A., Grünwald, T., Hari, P., Jans, W., Kellomäki, S., Loustau, D., Magnani, F., Markkanen, T., Matteucci, G., Mohren, G.M.J., Moors, E., Nissinen, A., Peltola, H., Sabaté, S., Sanchez, A., Sontag, M., Valentini, R. and Vesala, T. (2002), Evaluation of six process-based forest growth models using eddy-covariance measurements of CO2 and H2O fluxes at six forest sites in Europe. Global Change Biology, 8: 213-230.

Lantuit, H. (2016). What is permafrost? International Permafrost Association.

M. Syndonia Bret-Harte, Eugenie Euskirchen, Kevin Griffin, and Gaius Shaver. (2018). Eddy Flux Measurements, Tussock Station, Imnavait Creek, Alaska - 2016. Arctic Data Center. https://pasta.lternet.edu/package/metadata/eml/knb-lter-arc/20043/3.

Ma, Q., Jin, H., Yu, C., & Bense, V. F. (2019). Erratum to: Dissolved organic carbon in permafrost regions: A review. *Science China Earth Sciences*, *62*(4), 750–750.

Marrero, G. A. (2010). Greenhouse gases emissions, growth and the energy mix in Europe. Energy Economics 32, 1356–1363.

Oechel, W. C., Hastings, S. J., Vourlrtis, G., Jenkins, M., Riechers, G. and Grulke, N. (1993). Recent change of Arctic tundra ecosystems from a net carbon dioxide sink to a source. Nature 361, 520–523.

Pollard, W. (2018). Periglacial Processes in Glacial Environments. *Past Glacial Environments* 537–564.

Romanovsky, V., and Osterkamp, T. (2001). Permafrost: changes and impacts. NATO Science Series 76, 297-315

Schaefer, K., Zhang, T., Bruhwiler, L. and Barrett, A. P. (2011). Amount and timing of permafrost carbon release in response to climate warming. *Tellus B* 63, 165–180.

Segal, R. A., Lantz, T. C. and Kokelj, S. V. (2016). Acceleration of thaw slump activity in glaciated landscapes of the Western Canadian Arctic. Environmental Research Letters 11, 034025.

Ted Schuur. (2018). AmeriFlux US-EML Eight Mile Lake Permafrost thaw gradient, Healy Alaska. 2008-. Arctic Data Center.

Walker, G. (2007). A world melting from the top down. Nature 446, 718–721..

Walter C. Oechel, Allen S. Hope, and Douglas A. Stow. Large Area Estimates of Carbon Fluxes of Arctic Landscapes. Arctic Data Center.

The Connection Between Arctic Permafrost and Global Warming

Introduction:

Question

How do trends in Net Ecosystem Exchange (NEE) compare between continuous and discontinuous permafrost zones?

Background

- Tundra holds twice as much carbon as the atmosphere[1]
- Enhanced Greenhouse Effect promotes carbon release[2]
- Discontinuous permafrost melts from both top and bottom layers[3]

Gap

Tundra transition from sink to source but little understanding of how each permafrost type thaws throughout the years[4]

Hypothesis

Both discontinuous and continuous permafrost zones will show positive net ecosystem exchange (NEE) values over time. In comparison to continuous zones, discontinuous zones will release more carbon due to more plentiful ice cracks that promote thawing; also, discontinuous zones will respond to changes in climate more dramatically due sunlight penetration through ice cracks which warms deeper ice layers.

Methods:

Continuous and discontinuous zones obtained from Arctic Data Center

Variables Isolated: NEE and Collection Month

NEE outliers removed

Subset by discontinuous and continuous zones

Independent: collection month & permafrost type
Dependent: NEE

Linear regressions were run on each zone. ANOVA was then run

Results:

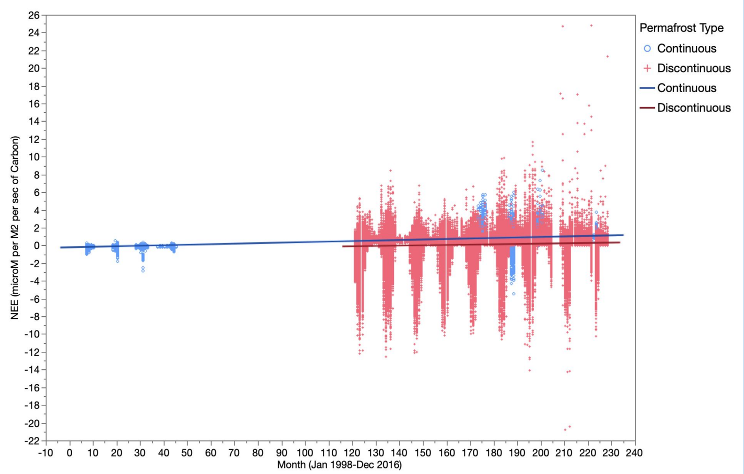

Figure 1. Net Ecosystem Exchange (NEE) of Continuous Permafrost and Discontinuous Permafrost Zones 1998-2016. Five sites of data were compiled, three sites of continuous and 2 sites of discontinuous and plotted together. Each dot represents a daily NEE measurement within a given month. Linear Regression, $R^2 = 0.003338$, $P < 0.0001$, type discontinuous estimate: 1.169×10^2, NEE= 0.003756x -0.5058, type continuous estimate: 0.1498, NEE= 0.005896x -0.1707. ANOVA, P< 0.0001.

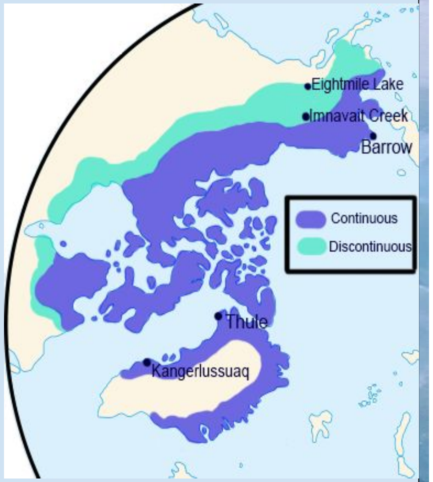

Figure 2. Continuous and Discontinuous permafrost zones in the Arctic region. Shown are the sampled sites in Greenland and Alaska. Continuous zones lie further centered than discontinuous zones due to ice stability differences.

- Discontinuous had a higher positive NEE estimate but a less steep NEE slope compared to continuous
- Relationship between Permafrost zone and NEE is statistically significant (P<0.0001)
- ANOVA showed that differences in NEE patterns between continuous and discontinuous zones are significant
- Results support published research that discontinuous zones are releasing more carbon than continuous[3]

Conclusion:

- Continuous permafrost climate response is faster than discontinuous seen by a steeper slope
- Greater carbon release for discontinuous zones is signified by a larger regression estimate
- Faster climate response suggests that continuous zones may soon resemble discontinuous zones

Future directions:

What factors impact the continuous permafrost's rapid response to climate warming?

References:

1.Webb, E., Schuur E., Edward, G., Natali, S., Oken L., Kiva, B., Rosvel, K., John, D., and Nickerson, N. (2016). Increased wintertime CO2 loss as a result of sustained tundra warming. JGR Biosciences *121*, 249-262.
2. Jain, P. (2003). Greenhouse effect and climate change: scientific basis and overview. Renewable Energy *3*, 403-420.
3. Romanovsky, V., and Osterkamp, T. (2001). Permafrost: changes and impacts. NATO Science Series *76*, 297-315
4.Tarnocai, C., Canadell, J.G., Schuur, E.A.G., Kuhry, P., Mazhitova, G., and Zimov, S. (2009). Soil organic carbon pools in the northern circumpolar permafrost region. Global Biogeochemical Cycles *23*, 1-11.
5. Background Picture:https://www.popsci.com/arctic-ice-melt-natural-climate-change/

Climate Change and Hurricane Effects on Florida Lakes Nutrient Levels

Background

- Global climate change is causing an increase in severity of hurricanes.[1]
- Productivity of lakes decrease following a major rain event and there's an increase in dissolved organic material.[2] It hasn't been shown how hurricanes affect nutrient levels.
- An excess of nutrients can cause eutrophication, decreasing lake productivity, biodiversity and an increase in lake toxicity.[3]

Research Question: How does the change in severity of hurricanes affect a lake's nutrient levels?

Hypothesis: As the intensity of tropical storms increase, there will also be an increase of nitrogen and phosphorus levels in the lakes following severe hurricanes.

Methods

1. Determine lakes to analyze from examining recent hurricane paths in the past 20 years from *NOAA*
2. Gather sample data of Nitrogen and Phosphorus levels in ug/L in Lakes Okeechobee and Istokpoga from *Florida Atlas of Lakes.*
3. Subset sample data to range from 1 year before and after a Hurricane event occurs
4. Graph sample data into a scatterplot with one lake and one hurricane event.
5. Run Pearson's R test to determine if there is a correlation among the hurricane events and nutrient levels of the lake.

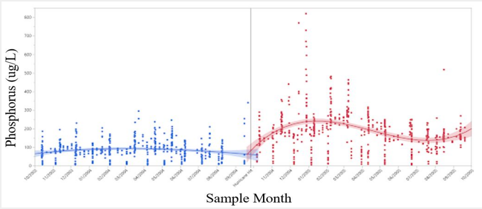

Figure 1: Phosphorus levels in Lake Okeechobee a year before and after Hurricane Jeanne. Prior to the hurricane, the R^2 value was 0.02 and the P-value was 0.0062. After the hurricane, the R^2 was 0.14 and the P-value was less than 0.0001.

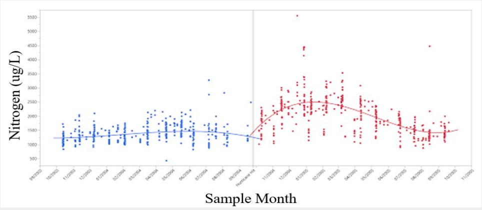

Figure 2: Nitrogen levels in Lake Okeechobee a year before and after Hurricane Jeanne. Before the hurricane, the R^2 value was 0.11 and the P-value was less than 0.0001. After the hurricane event, the R^2 value was 0.19 and the P-value was less than 0.0001.

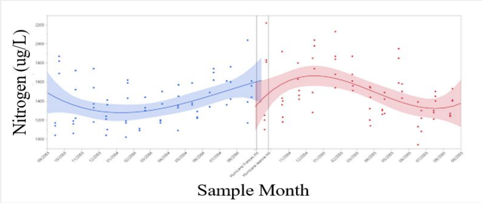

Figure 3: Nitrogen levels in Lake Istokpoga a year before and after Hurricane Frances. The R^2 value prior to the hurricane was 0.18 while the R^2 value following the hurricane was 0.02. The P value before the hurricane was 0.0037, and the P value following was 0.6552.

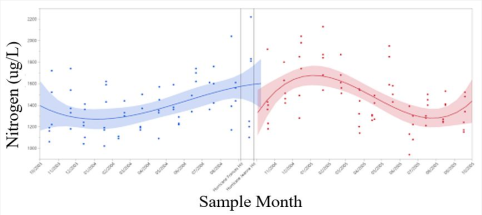

Figure 4: Nitrogen levels in Lake Istokpoga a year before and after Hurricane Jeanne. Before the hurricane, the R^2 value was 0.21 and the P-value was 0.0013. After the hurricane, the R^2 value was 0.31 and the P-value was less than 0.0001

Results

- A general increase of nutrient levels following hurricane events.
- Lake Okeechobee experienced an exponential increase in phosphorus and nitrogen following Hurricane Jeanne.
- Analysis of Lake Okeechobee following Hurricane Wilma did not yield a significant change.
- Nitrogen levels in Lake Istokpoga were the only nutrient to experience a statistically significant increase following both hurricane events.

Conclusion

- Rainfall deposition caused by a major weather event, such as a hurricane, causes an increase in nutrient levels in the lakes affected.
- The nutrients affected by the hurricane event could be influenced by the size of the affected lake.
- The nutrient levels that will change following a hurricane event increase exponentially and then return to a level slightly above the nutrient level exhibited by the lake in the year before the hurricane event.
- Once water nutrient testing can be performed daily more accurate results can be obtained.

1. Webster, P., Holland, G., Curry, J., and Chang, H. (2005). Changes in tropical cyclone number, duration, and intensity in a warming environment. *Science* 309, 1844-1846. 2. Sadro, S., and Melack, J. (2012). The effect of an extreme rain event on the biogeochemistry and ecosystem metabolism of an oligotrophic high-elevation lake. *Arctic, Antarctic, and Alpine Research* 44, 222-231. 3. Carpenter, S. R. (2005). Eutrophication of aquatic ecosystems: Bistability and soil phosphorus. *Proceedings of the National Academy of Sciences*, *102*(29), 10002–10005. Doi: 10.1073/pnas.0503959102
